# Hybrid Carbohydrate–Lipid Nanocarriers: In Vitro Efficacy Gene-Rated by Association of UV-Absorbers and Raspberry Polyphenols Rich-Fraction

**DOI:** 10.3390/ph18010016

**Published:** 2024-12-26

**Authors:** Nicoleta Badea, Diego Samayoa, Alina Moroşan, Cristina Ott, Ioana Lacatusu

**Affiliations:** Faculty of Chemical Engineering and Biotechnology, National University of Science and Technology POLITEHNICA of Bucharest, Polizu No 1, 011061 Bucharest, Romania; nicoleta.badea@upb.ro (N.B.); dsamayoaiq@gmail.com (D.S.); alina.morosan@upb.ro (A.M.); cristina.ott@upb.ro (C.O.)

**Keywords:** hyaluronic decorated lipid nanocarriers, dual Raspberry-UV absorbers, antioxidant action, photoprotective effect, nanocarrier conditioned creams

## Abstract

**Background/Objectives:** The study aims to investigate an improved version of lipid nanocarriers (NLCs) (formulated with functional coconut butter and marula oil) by designing hyaluronic acid (HA) decorated NLC co-loaded with dual UVA (butyl methoxy dibenzoyl methane, BMDBM), UVB absorbers (ethyl-hexyl-salicylate, EHS) and a Raspberry rich polyphenols fraction (RPRF) for development of more natural NLC-based to-pical formulations. **Methods**: Quality and quantitative attributes of classic- and HA-NLC have been assigned based on particle size, electrokinetic potential, encapsulation efficiency, spectroscopic characteristics, and high-resolution mass spectrometry. To establish the performance profile of antioxidant activity, release of active substances, sun blocking action, and photostability, in vitro studies were conducted. **Results**: NLC with an average size of ~150 nm and zeta potentials < −39.5 mV showed 80% and 93.1% of encapsulation efficiency for BMDBM and EHS, and up to 83% for natural RPRF. A long-lasting release of absorbers, with a maximum cumulative release of 2.1% BMDBM and 4.6% EHS was detected. NLC-UV Abs-RPRF-HA assured 72.83% radical scavenging activity. The IC_50_ for HA-NLC-UV Abs-RPRF was 6.25-fold lower than NLC-UV Abs-HA, which reflects the greater free radical scavenging action. The conditioned NLC–UV Abs-RPRF-HA cream was able to provide a sun protection factor value of 52 and UVA-PF value of 81, which underlines an impressive removal of both categories of UVA and UVB radiation. A significant photoprotective upregulation, four-fold for the topical formulation with NLC-UV Abs-RPRF-HA, resulted after a simulated irradiation process. **Conclusions**: HA decorated-NLC-conditioned creams might provide a useful platform for developing na-tural and sophisticated dermal delivery systems, for influencing skin permeability, and for synergistically imparting antioxidant and photoprotective actions to cosmetic pro-ducts.

## 1. Introduction

Applications of nanotechnology and plant resources, whether vegetable oils or herbal extracts, in topical formulations have emerged, providing numerous benefits in the development of advanced cosmetic prototypes. There is a growing need for safe and effective topical sunscreens containing body-friendly actives capable of counteracting the dangerous effects of UV-A and UV-B radiation [1]. UV-A radiation (320–400 nm) penetrates deep into the skin, reaching the dermal fibroblasts, thus affecting the connective tissue and increasing the production of harmful reactive oxygen species, ROS [2]. Wavelengths in the UVB region (~320 nm), although less frequent, are more invasive, because they are mainly absorbed by epidermal cell components, such as proteins or DNA. Both categories of UV-A and UV-B radiation can cause serious damage to human health, such as premature ageing of the skin, sunburn, or chronic consequences, such as skin cancer [3]. Sunscreen agents (organic UV absorbers) have great advantages, but at the same time they can undergo photodegradation under exposure to sunlight or can penetrate through the skin layers and be distributed in the body. To be effective, UV filters must be retained on the upper surface of the skin as a protective film, where they should remain photostable throughout the entire period of UV exposure. Skin penetration and/or light-induced decomposition are undesirable events, as they can form photodegradation products that cause skin irritation (photo-dermatosis), including phototoxicity and allergic reactions [4]. Unfortunately, according to animal and clinical studies, the skin penetration of UV filters from topical products and their systemic absorption is of concern because they result in endocrine disruption [5]. A recent announcement, published by the Food and Drug Administration (FDA) in its regulations of 2019, has urged the safe use of UV filters and questioned the skin permeability and toxicity that may possess hidden risks for humans [3]. Theoretically, UV absorbers’ activity should be restricted to penetrating the skin or to within the upper layers of the stratum corneum to avoid adverse systemic effects. The most popular UV filters are lipophilic molecules with relatively low molecular weight, which makes them able to penetrate trans-dermally and be absorbed systemically.

An example of an organic UV absorber widely used in sunscreen products is butyl-methoxy dibenzoyl methane, BMDBM (4-tert-butyl-4′-methoxydibenzoylmethane). BMDBM, also known by the commercial names Parsol 1789 or Eusolex 9020, is a strong UVA protection filter that exists in an enol and keto tautomeric form [6]. Photodegradation products of avobenzone could be responsible for photoallergic and cytotoxic reactions, causing allergies and contact dermatitis [7]. Therefore, although sunscreens are formulated to achieve maximum efficiency and measures are taken to promote photo-stabi-lity, there is still a need to develop innovative skin-based formulations. Multiple efforts are thus being devoted to achieving safer and appealing suncare products, and the encapsulation of UV filters in different micro- and nanostructured structures for dispersion in cosmetics, is one of the most popular strategies.

As an alternative to reduce photoinactivation and skin penetration of UV filters, while maintaining the efficacy and safety of photoprotectors, a system based on nanostructured lipid carriers (NLC) loaded with organic filters has been proposed [8,9]. Nanostructured lipid carriers are one of the encapsulation approaches suitable for utilization in topical preparations. The most important features that have made NLCs popular are their ability to enhance the photostability of photolabile UV absorbers due to their solid lipid matrix [10] and the sustained release over time of UV active, which reduces skin irritation at high UV-absorber concentration [6]. Importantly, NLCs may scatter UV-A and UV-B radiation in synergy with UV absorbers [11], while UV screening potential can be doubled compared to conventional emulsions. In addition, the NLC occlusion effect has also contributed to their popularity, since they reduce skin water loss, increasing hydration. The reduced particle size of NLCs prevents them from penetrating skin and allows an appropriate skin adhesive film formation, which enables comfortable skin application [12]. In recent years, a considerable number of reports have been published on the application of NLC for enhancing the protective efficacy of photo-unstable UV absorbers. One of the newest pieces of research was by the Martins group, which studied the incorporation of butylmethoxydibenzoylmethane/BMDBM together with rutin [13]. Other approaches, such as the incorporation of natural antioxidants, have been scientifically proven to improve UV absorbers’ photostability and photoprotective activity in lipid nanocarrier formulations [14]. For instance, kenaf seed oil-based NLC loaded with diethyl-amino-hydroxy-benzoyl hexyl benzoate (DHHB), ethyl-hexyl-triazone (EHT) and tocotrienol demonstrated high efficiency in providing compatibility for UV-filter encapsulation [11]. Furthermore, NLC-based *Acrocomia aculeata* bocaiuva oil containing avobenzone and octocrylene was found in antioxidant synergy [12]. Generally, antioxidants in vegetable oils have been studied, in order to reduce the concentrations of UV filters in sunscreens, maintaining or increasing their efficacy, or even obtaining photostable systems that are safer for the consumer [2,15]. Bearing in mind these strategies, NLCs could be seen as excellent delivery systems for organic UV-absorbers because, besides their inherent biocompatibility, NLCs are also able to host active compounds with antioxidant properties, improving the skin care assets of the formulation or the photostability of UV-Absorbers [6].

Tuneable skin penetration is a particularly relevant feature when developing sunscreen products, once these preparations are repeatedly applied on skin areas. To influence the cutaneous features, the covering of NLC with biocompatible polymers represents a suitable approach. Hyaluronic acid (HA) is a natural polysaccharide of D-glucuronic and N-acetylglucosamine distributed in the connective and epithelial tissues. HA has been characterized by multiple pharmacological actions in skin repair, anti-ageing, tissue regeneration, anti-inflammatory activity, and wound healing [16]. HA is also demonstrated to stimulate collagen production [17], promote the moisturizing power of skin [18], relieve wrinkles, and improve hydration of skin [19]. Combined with its biocompatibility, non-immunogenicity, bio-adhesive property, and tuneable viscoelasticity [20], HA associated with NLC presents a relevant subject for study, with positive consequences for the development of advanced dermato-cosmetic bioproducts. HA-NLCs are of paramount importance in the treatment of skin disorders and represent a hot-spot in the research and development of skin care products. The design of NLC systems coated with HA has been reported to reduce the high in vivo dosage and weak targeting ability of antitumor paclitaxel [21], while HA-coated Teriflunomide-NLC demonstrated a suitable role in the oral management of rheumatoid arthritis [22]. An effective transdermal local anaesthetic therapy and improved antinociceptive effect have been proved for HA-modified NLC loaded with bupivacaine [23]. In other related studies, HA-modified NLC loaded with a natural flavonoid, kaempferol or curcumin, was constructed to assess the antitumor effect on A549 cells [24] and to investigate the anti-inflammatory effect on Coco-2 cells [25].

Several lipid-based nano-cosmetics are commercially available, at different phases of clinical trials [5], but none approaches the strategy of coating NLC with hyaluronic acid. The development of effective and safe products requires high photostability to preserve UV-blocking capacity and minimal percutaneous permeation, in order to retain activity in the upper skin layers. HA-decorated NLC can respond to these requests, as they are smart and dual-response pharmaco-cosmetic nanocarriers, because, besides their ability to mimic the endogen lipid environment found in the corneum stratum, they are also able (i) to be excellent vehicles for actives with antioxidant and photoprotective properties, thus improving skincare assets [6]; (ii) to insert into a final cosmetic nano-formulation a multiple-responsive effect, due to HA itself, but also to the hydrophilic natural ingredients retained by this carbohydrate shell; (iii) to assure enhanced skin penetration due to affinities with HA epidermal keratinocytes receptors; (iv) to exhibit an improved ability to facilitate deposition of actives throughout the skin in favor of inflammation prevention and that of degenerative skin diseases [19].

The novelty of the present study is the development of an improved version of classic NLC, using the design of HA-decorated NLC-containing synthetic and natural actives to develop more natural and functional nanostructured based topical formulations containing body-friendly nano-actives capable of counteracting the dangerous effects of ra-dical oxygen species and UV-A and UV-B radiation. The unique combination of functional lipids from coconut butter, marula oil for designing HA modified-NLC able to host couple of UV-Absorbers (e.g., butyl methoxy dibenzoyl methane, BMDBM and ethyl-hexyl-Sali-cylate, EHS) and natural Raspberry polyphenol-rich fractions (RPRF) is worth evaluating in their performance in the reduction of dangerous UV absorbers and to complement the entire pharmaco-cosmetic benefits of specially designed carbohydrate–lipid nanocarrier based-creams. The use of bioactive lipids, such as marula oil (MO) and coconut butter (CB), is particularly important due to their functional lipids having been found to reduce skin ageing and neutralize photogenerated reactive species. MO comes from the Marula Tree (*Sclerocarya birrea*, originating from Southern Africa), with a high content of toco-pherols and phytosterols, which not only enhance its antioxidant activity but also give it anti-inflammatory and skin-regenerating properties. Triglycerides, fatty acids, phytoste-rols, and carotenoids are among the bioactive compounds usually found in MO [26], most of these with antioxidant properties and impressive effects in protecting the skin against ROS. MO assures hydrating, moisturising and emollient properties [27]. According to Komane et al., the safety and efficacy of MO, i.e., non-irritancy potential, hydrating and occlusive effects, have been shown from a clinical perspective; marula oil was classified as safe for use in cosmetic products, where minimal absorption occurs [28]. Cocoa butter (CB) contains a high number of medium-chain triglycerides, it is a source of vitamin E, which makes it well suited as a cosmetic ingredient in skincare products. The lipids present in cocoa butter create a protective barrier that retains moisture and prevents skin from drying out. CB is recognized for its benefits, including protecting the skin from sun da-mage and being a primary part of many topical treatments for eczema, dermatitis and skin healing [29,30].

Thanks to these beneficial and functional lipids, the hybrid HA-decorated NLC contributes to protection against degenerative skin diseases, assuring hydrating, moisture-zing, antioxidant and anti-inflammatory properties. In addition to vegetable lipids, MO and CB, the co-presence of other natural components, such as vegetal polyphenol fractions, in sunscreen formulations can be an alternative for the reduction of UV classic absorbers; they may act as antioxidant enhancers and can improve the photoprotection offered by UV-absorbers. The main phytochemicals found in Raspberry fractions, i.e., phenolic acids and flavonoids (quercetin, catechins, caffeic acid, acid sinapic acid, ferulic acid, chlorogenic acid); anthocyanins (cyanidin-3-glucoside, cyanidin-O-glucuronide, cyanidin-3-rutinoside, delphinidin-3-rutinoside); and ellagitannins (Sanguiin H-6, Lambertianin C), are efficient anti-inflammatory and antioxidant agents, may inhibit lipid pero-xidation [31], and have been reported to achieve improved clinical scores for wrinkles, suppleness, firmness, roughness and skin hydration [32]. To the best of our knowledge, there has been no attempt to combine these distinct elements: 1. the concomitant association of UV-absorbers, Raspberry polyphenol-rich fractions and NLC, aiming to create a synergistic photoprotection and antioxidant effect; 2. a surface decoration of NLC with a hyaluronic polymer to reinforce the moisturizing, regenerating and healing effect on the skin.

## 2. Results and Discussion

Since more and more patients are being diagnosed with skin cancer each year, this research addresses a major question of importance to pharmaco-cosmetic scientists: how can nanocarrier systems play a pivotal role in maximizing the photoprotective potential of sunscreen and antioxidant efficacy, while minimizing organic filter concentration and skin penetration? The compositions and structures of nanocarriers are extremely varied; however, those prepared using biodegradable and biocompatible lipids and carbohydrates are preferred, since they play a key role in chemical and physical stability, and thus, in photostability. Different arrangements, whether compartmented or in a matrix structure, will reflect the performance and success of the encapsulated UV-absorber and natural Raspberry polyphenol-rich fraction.

### 2.1. Optimization of Nanocarriers Based on Particle Size, Polydispersity Index, and Electrokinetic Potential

The chemical composition, mainly of emulsifiers, and the ratio of HA polysaccharides applied to modify the surface of lipid nanocarriers are determining elementary factors in determining the characteristics of newly hybrid lipid nanocarriers. Thus, several categories of blank NLC with a blend of marula oil, coconut butter, and glycerol monostearate, at different ratios between Lecithin and Tween 20, have been prepared. The changes in particle size, polydispersity, and electrostatic potential due to different hyaluronic acid/ionic emulsifier ratios in each formulation were evaluated. According to Table 1, there were statistically significant differences in the mean particle size, ranging from 132 nm to 189 nm (Table 1). Interestingly, HA coating did not increase the Z_ave_ values of NLC-1/2/3, the average diameter values being even lower in certain cases, for instance, Z_ave_ = 139 ± 1 nm for NLC-3 vs. Z_ave_ = 137 ± 2 nm for NLC-3-HA-*a*. This behaviour may suggest a favorable accommodation of the carbohydrate anion among amphiphilic and polysorbate-type non-ionic surfactant shells. In addition, the particle distribution represented by the values of PDI was lower than 0.2 for most NLC formulations, suggesting a narrow size distribution.

Regarding the surface charges of NLC without surface HA modifier, as the content of Lecithin decreased, the values of the zeta potential underwent slight changes (Table 1), in the sense of decreasing the negative surface charge. The surface coating with HA led to a minor decrease in the surface charge of the NLC, the values being within the range of colloidal stability, i.e., between −39.5 mV and −56.6 mV, which ensures the avoidance of coalescence and flocculation phenomena [33]. Thus, the HA polysaccharide applied to modify the surface of lipid nanocarriers can maintain electrostatic stability and provide steric stability (by repulsion).

A comparative evaluation of the size and stability parameters of NLC with and without surface HA modifier reveals the best behaviour of the NLC-3, in which a 3:1 ratio between amphiphilic emulsifier and hyaluronic acid was used. This ratio is considered optimum because sufficient positive charges are provided for the electrostatic attraction of the hyaluronic acid anions while avoiding the potential of liposome formation (when a high content of lecithin is used).

**Figure 1:**
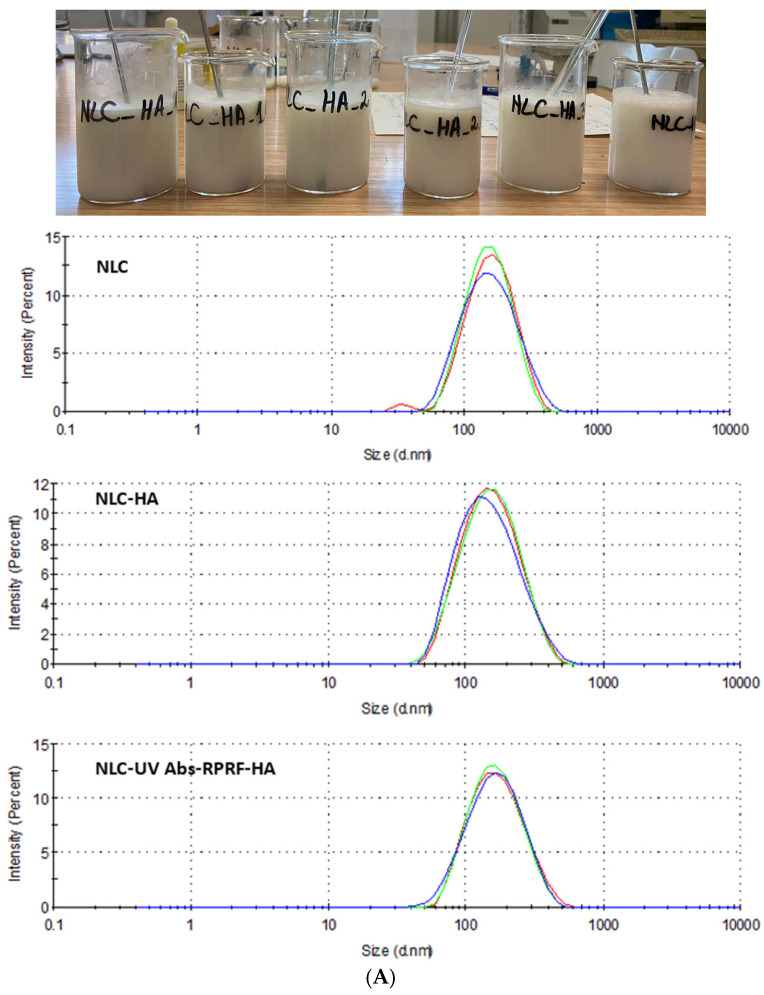

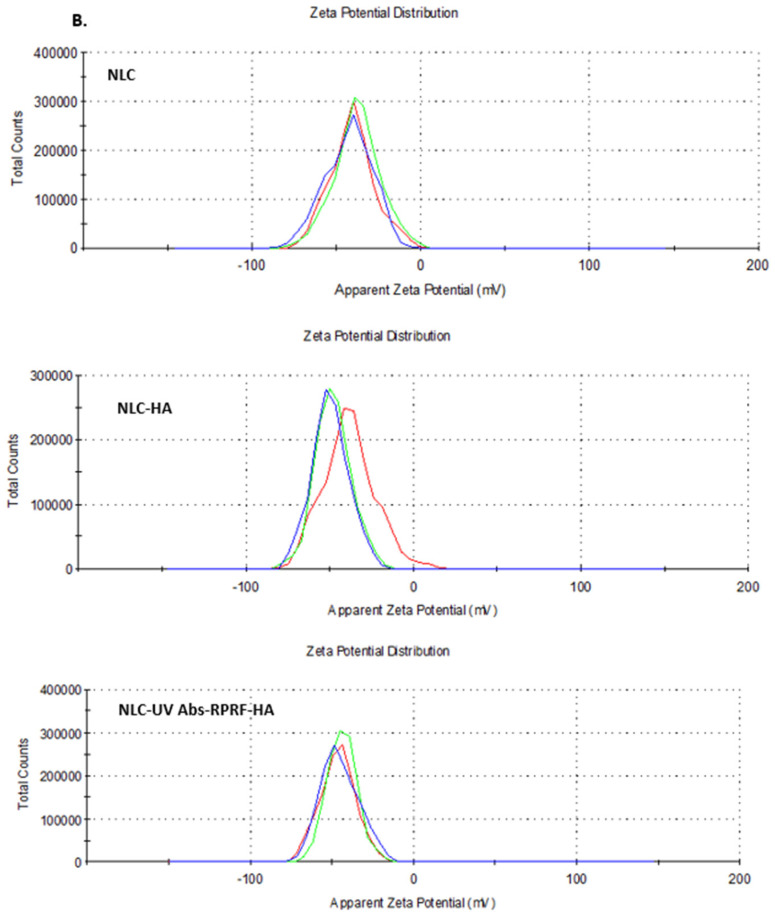
Size distribution (**A**) end electrokinetic potential distribution (**B**) of selected blank- and UV-Absorbers and Raspberry polyphenol fraction loaded NLC with/without surface HA modifier.

As such, the NLC-3 formulation was subjected to the encapsulation of the two UV-A and UV-B absorbers, but also of a mixture of BMDBM, EHS, and Raspberry Polyphenol Rich Fractions (RPRF). It is worth noting that the loading of the actives led to higher main diameters for both conventional and hybrid hyaluronic modified-NLC (Figure 1), ranging from 150 ± 3 nm, with PDI = 0.156 ± 0.001 (for NLC-UV absorbers–Raspberry-HA *a*) to 167 nm ± 1 nm with PDI = 0.187 ± 0.013 (for NLC-UV absorbers–Raspberry). A difference of approx. 15–20 nm in the Z_ave_ of NLC-HA compared to NLC-HA-UV absorbers–Raspberry was recorded when they were subjected to DLS analysis (Figure 1). These findings confirm the extension of nanocarriers due to the capturing of synthetic and natural principles. An elevated mean diameter after co-encapsulating willow bark extract together with octocrylene and avobenzone filters has been reported by our group [34]. Strongly electrone-gative surfaces have been maintained by entrapment of BMDBM and EHS absorbers and Raspberry Polyphenol Rich Fractions into NLC with/without surface HA modifier. Determined zeta potential values were −44 ± 1 mV, for NLC-UV Abs-Raspberry and −45 ± 1 mV for those NLCs covered by hyaluronic acid, suggesting no risk of coagulation phenomena or phase separation, and increased physical stability.

### 2.2. Qualitative and Quantitative Attributes of HA Decorated-NLC Loaded with Dual UV-Absorbers—Raspberry Polyphenol Fraction

**Figure 2:**
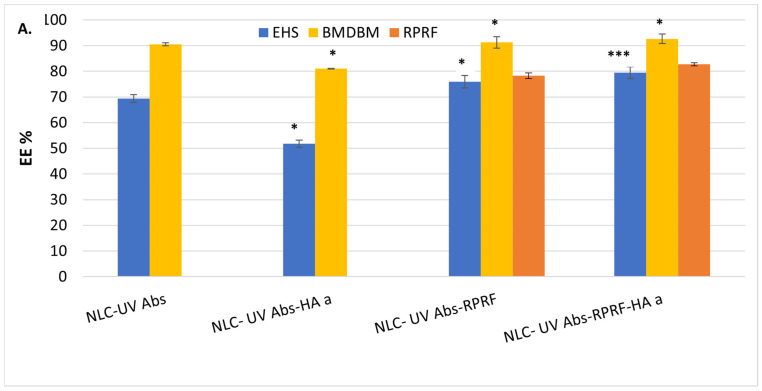

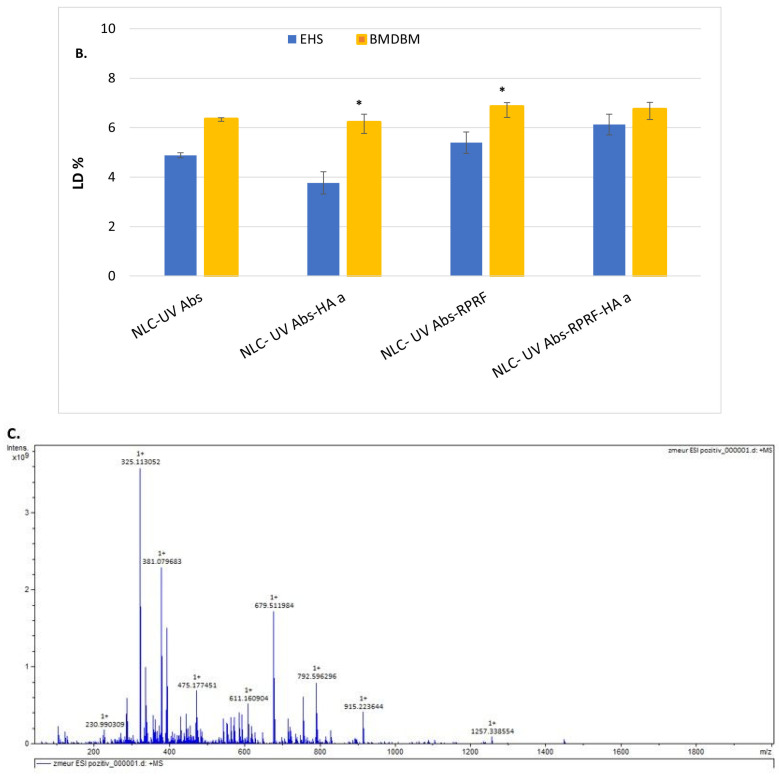
Quantitative attributes of HA decorated-NLC loaded with UV Absorbers and the dual UV-Absorbers-Raspberry polyphenol fraction: entrapment efficiency (**A**), loading capacity of lipid nanocarriers (**B**), and FT-ICR MS/MS of Raspberry polyphenol fraction (the measured mass and calculated mass, *m*/*z* are included in Supplementary Materials, Table S1) (**C**). All experiments were performed in triplicate. * *p* < 0.05; *** *p* < 0.0005; Data are expressed as mean ± SD, *n* = 3, NLC-UV Abs vs. other groups.

The imperfections generated during the association of marula oil and coconut butter distort the formation of a perfect lipid core, providing nano compartments within the solid matrix, ideal for entrapping lipophilic BMDBM and EHS absorbers and minimizing their potential expulsion. According to the quantitative study, high encapsulation efficiencies were determined for BMDBM (between 81% and 92.7%), while the capture of EHS was a little more difficult, the efficiencies for EHS absorber not exceeding 80% (Figure 2). The loading difficulties encountered by EHS can be correlated with a potential orientation of its hydrocarbon tails on different axes compared to the salicylate aromatic cycle from BMDBM, which would make it difficult to accommodate inside the lipid network’s imperfections. The result of EHS encapsulation into NLC obtained by Lacatusu et al. was 77% [35]. An aspect worth mentioning is the influence of the Raspberry polyphenol-rich fraction that led to a slight improvement in the EE% of both BMDBM and EHS absorbents. This aspect can be explained by the protective role of hydrophilic phenolic phytoconstituents from Raspberry housed in the surfactant coating; these no longer allow (hinder) the release of UV absorbers.

The loading capacity of the UV absorbers is determined by the amount of lipid matrix, up to a maximum of 10%, that can be achieved. The lipid matrix of HA-modified NLC ensured a maximum of 92.7% ± 1.83 of encapsulation efficiency and an appropriate loading capacity for BMDBM with a maximum of 6.74% ± 0.28 (Figure 2). The high loading capacity is due to the ratio of UV absorbers to lipid content, which is low enough to achieve maximum encapsulation. Additionally, the incorporation of marula oil into solid lipids can lead to a disordered lipid structure, leaving enough space to accommodate the active absorbers [36], and intentionally used for higher loading capacity.

The high encapsulation of the UV filter enables the controlled release of the active ingredients to prevent the dangerous photodegradation of BMDBM. Furthermore, the high encapsulation efficiency of UV-A and B absorbers is beneficial in reducing skin irritation due to the avoidance of direct contact between synthetic actives and the skin surface.

To quantify the ability of HA-decorated NLC-UV Abs to host RPRF, the Raspberry fraction was analysed by high-resolution mass spectrometry FT-ICR–MS (positive ionization) and Folin-Ciocâlteu spectroscopy. The *m*/*z* identified masses of the main phenolic acids, catechins, cyanidin, and delphinidin derivatives were in agreement with the *m*/*z* masses of the protonated ions, ESI+ reported in the literature [37,38]. The monoisotopic MS bands of the compounds were identified at 181.0497 *m*/*z* (caffeic acid [M+H]^+^), 181.0708 *m*/*z* and 203.0527 (myo-inositol [M+H]^+^ and [M+Na]^+^, respectively), 195.0652 *m*/*z* and 217.0481 *m*/*z* (ferulic acid [M+H]^+^ and [M+Na]^+^, respectively), 377.0844 *m*/*z* (chlorogenic acid [M+H]^+^), 225.0759 *m*/*z* and 247.0578 (sinapic acid [M+H]^+^ and [M+Na]^+^, respectively), 291.0863 *m*/*z* (catechin [M+H]^+^), 449.1080 (cyanidin-3-glucoside M^+^), 595.1661 *m*/*z* (cyanidin-3-rutinoside M^+^), 465.1091 *m*/*z* (delphinidin-3-glucoside M^+^), 611.1609 *m*/*z* (delphinidin-3-rutinoside M^+^) and 479.0795 *m*/*z* (quercetin-3-glucoside [M+H]^+^). Measured mass (*m*/*z*) and calculated mass (*m*/*z*) values (Bruker Compass Data Analysis software) are found in the Supplementary Materials (Table S1).

The content of polyphenols in the Raspberry fraction was 4.52 ± 0.32 mg GAE/g dried Raspberry fraction, which is similar to the results of the determinations carried out on 4.45 ± 0.219 mg GAE/g dried Raspberry extract [39]. The hydrophilic phytochemicals from RPRF were successfully hosted in the lipid nanocarriers, most likely distributed in the outer shell of the surfactants, as evidenced by the values over 80% of the entrapment efficiency. A small improvement of EE% was reported in the case of hyaluronic acid decorated-NLC, 82.7 ± 1.83% EE; this slight increase can be explained by the protective role of the HA surface modifier. The creation of weak bonds between Raspberry polyphenols and the functional groups of hyaluronic acid may also be a result of the more efficient entrapment of RPRF in HA decorated-NLC than in conventional NLC.

### 2.3. FT-IR Characterisation

**Figure 3:**
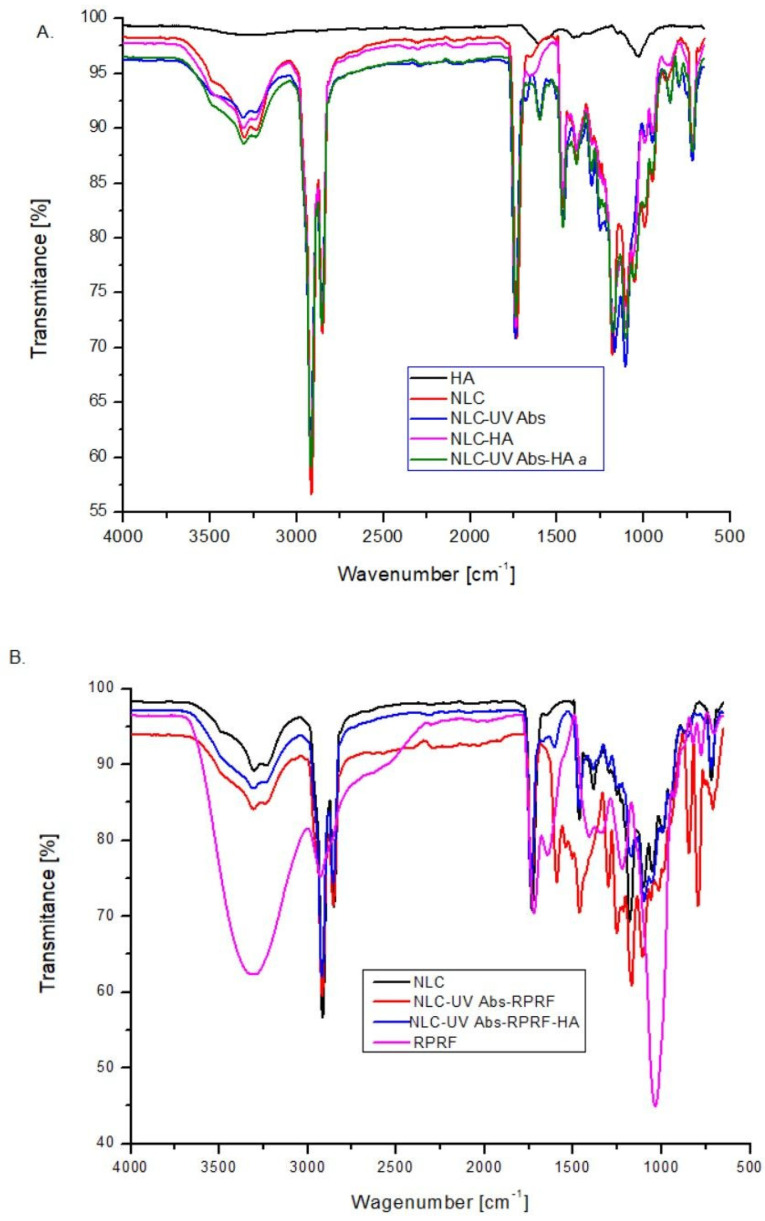
FT-IR spectra of NLC loaded with UV-A and-B Absorbers (**A**) and NLC co-entrapping UV Absorbers and RPRF (**B**) with and without decorated HA.

The FT-IR analysis of conventional lipid nanocarriers’ entrapping active principles and hybrid hyaluronic-decorated lipid nanocarriers co-loaded with UV-Absorbers and Raspberry polyphenol-rich fraction was performed to observe if there were potential interactions on the surface of the nanocarrier and to visualize the presence of Raspberry extract and hyaluronic acid on the outer surface of the NLC. Multiple strong bands were discovered by FT-IR (Figure 3). As displayed in Figure 3, the band centred around 3240 and 3310 cm^−1^ denoted the stretching of the multiple –OH groups and N–H from N-acetyl side chain of HA, from polyphenol rich fractions and Lecithin. The FTIR spectrum showed distinct bands at 2920 and 2850 cm^−1^ characteristic of the C–H stretching vibration (aliphatic CH asymmetric and symmetric stretch) [40].

As can be seen from ATR–FT-IR (Figure 3A), there are no differences between the IR spectra of the NLC categories that encapsulate only UV absorbers, which denotes the efficient encapsulation of the two sunscreens, BMDBM and EHS, in the lipid core. The bands observed in IR spectra of NLC-UV Abs and NLC-UV Abs-HA from around 1740 cm^−1^ were related to the C=O stretching, while that from 1060 cm^−1^ could be assigned for C–O–C of glucuronic acid of HA. 

In the NLC with a surface hyaluronic acid modifier, the stretching bands at 1657 and 1421 cm^−1^ are ascribed to the C=O stretching vibration, and those from 1520 cm^−1^ (amide II) attributed to C–N stretching and N–H bending vibration [37]. Other HA characteristic bands which appeared at 1617 cm^−1^ are assigned to the asymmetric COO^−^ group, whereas the symmetric carboxylate vibration band was evident at 1409 cm^−1^. The bands at 1175 and 1106 cm^−1^ correspond to –^+^NH= and C–O–C, respectively. These results agree with the previous reports of Zeail et al., who developed hyaluronic acid-coated lipid nanocarriers loaded with teriflunomide [22].

Note the slight shift of the band from 1106 cm^−1^ for NLC-UV absorber formulations (assigned for C–O stretch) to 1102 cm^−1^ in the NLC coloaded with UV absorbers and raspberry fraction, due to the involvement of hydroxyl groups in the hydrogen bonds [41].

The corroborating of spectral information reflects that no chemical interactions occurred among the NLC ingredients and agrees with the previous reports of Feng et al. (2025), who obtained hyaluronic acid decorated NLC-curcumin [25].

Figure 3B displays the comparative ATR-IR spectra of NLC, which co-entrap BMDBM, EHS, and Raspberry Polyphenol Rich Fraction/RPRF. The stretching of the C=C–C aromatic bonds from Raspberry fraction (especially tannins) are recognised in the region 1610–1440 cm^−1^ and for C–O stretching at 1368–1157 cm^−1^ and 1031–1023 cm^−1^. The bands located around 1026–1030 could also be assigned to the δ_C–O–C_ from glycosylated anthocyanins. The band centred between 1280 and 1370 cm^−1^ can be attributed to d of –O–CH and C–OH from the glycosidic part of flavonoids [38]. The region between 1722 and 1700 cm^−1^, corresponds to the C=O stretching of tannins, especially derivatives of gallic acid. The flavonoid absorption from regions between 1361 and 1340 cm^−1^ and 1284 and 1283 cm^−1^ is due to the C–O of pyran, typical of flavonoid C-rings [38].

In addition, the band from 1560 cm^−1^ encountered in the spectra of NLC-UV Abs-Raspberry could be attributed to benzene ring unsaturation, attributed to the aromatic hydrocarbons of phenolic acids, caffeic acid, gallic acid, and glycosidic cyanidins present in Raspberry Polyphenol Rich Fraction [42]. The observation of these bands in all the NLC-UV Abs-Raspberry indicated that the polyphenols had successfully been captured predominantly in the outer shell, and not inside the lipid core. Karimi et al. reported that major bands of turmeric extract had disappeared in turmeric-loaded NLC, which showed the entrapment of turmeric extract into the lipophilic core of NLC [43].

### 2.4. In Vitro Release Studies of BMDBM and EHS

**Figure 4:**
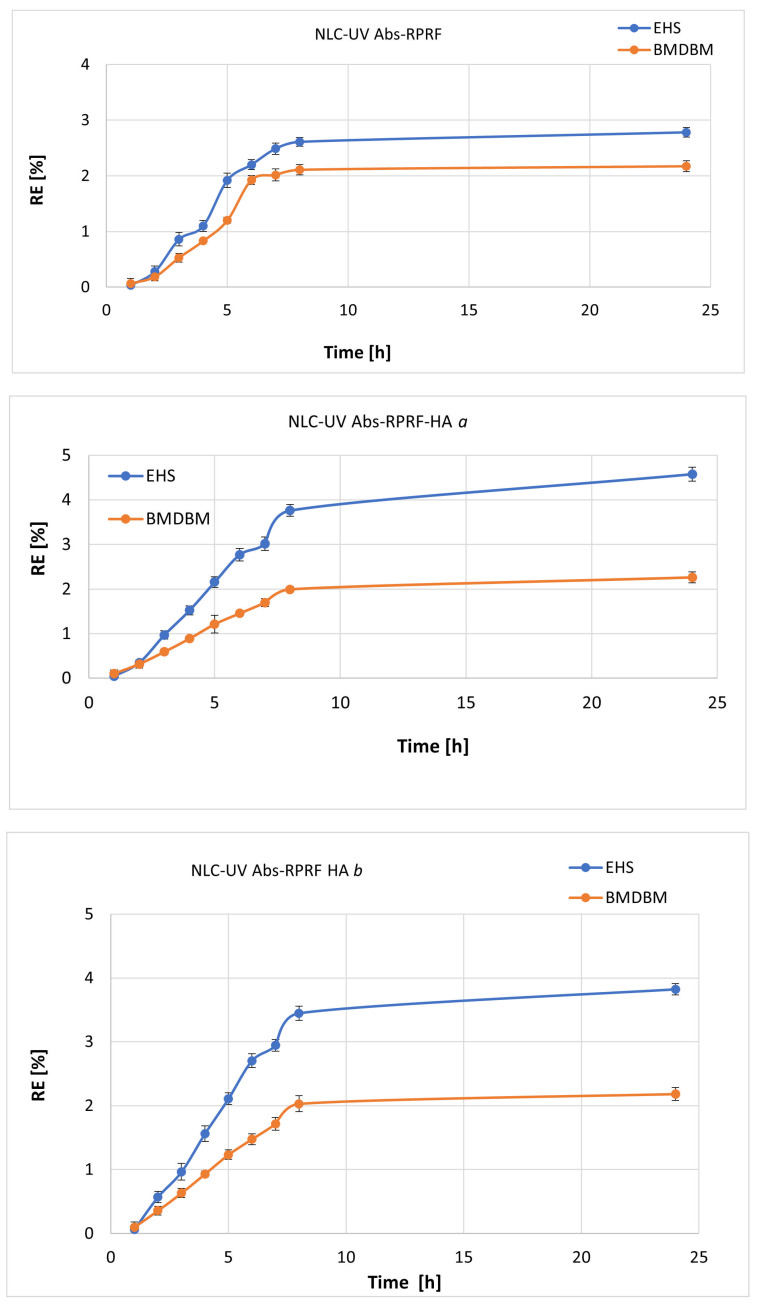
In vitro UV absorbers’ release from conventional and hybrid hyaluronic modified NLC.

The skin permeation of UV filters from sunscreen can increase the risk of toxicological reactions and affect the function of cosmetic formulas from UV radiation protection. The in vitro release profile of conventional and carbohydrate/HA-covered NLC-containing UV Absorbers (BMDBM and EHS) and RPRF were evaluated at up to 24 h of analysis. From the release profile indicated in Figure 4, all the NLCs showed a similar release pattern with a gradual release of both BMDBM and EHS filters, without detecting a burst release of the UV-A and -B absorbers, with <5% cumulative release after 24 h of study. This can be beneficial; a slower release of UV absorbers indicates a better retention capa-city of the active substances in the delivery system. This can be advantageous if a gradual and long-lasting release of sunscreens is desired. Thus, the active substances are released in a controlled manner over a longer period, ensuring constant and durable UV photoprotection.

The comparative in vitro release studies of the two UV-A and B absorbers from conventional-NLC and carbohydrate covered-NLC showed that, although both followed a gradual release, EHS is released more quickly than BMDBM from the delivery system. A slow release of UV absorbers (as determined for BMDBM absorber) may indicate, at first view, a greater solubility of the UV absorber/BMDBM in the delivery system and, on the other hand, a more limited mobility of the absorber to pass through the lipid network as a result of large molecular weight. A similar result was reported by Lacatusu et al., in that BMDBM cannot easily pass through the Franz membrane due to the high molecular weight of the UV absorbers [34]. Besides this, the faster release encountered in the case of EHS can be associated with a capture of an appreciable amount of EHS absorber in the nanocarrier shell, because of its structural maladaptation in terms of accommodation in the imperfections of the lipid core. The so-called “mismatch” of the EHS molecule in the lipid core network could also explain the slightly lower encapsulation efficiency of EHS (according to the previous entrapment determination results).

An aspect that deserves to be mentioned is the influence of the carbohydrate coating of NLC on the in vitro absorber’s release. According to the results summarized in Figure 4, the covering of NLC with HA accelerates the release of EHS and almost does not influence the release of BMDBM. For example, after 24 h of in vitro release study, in all tested delivery systems, the cumulative release of BMDBM remained almost constant, approximately 2.1%, while the cumulative release of EHS did not exceed 4.6% for NLC-UV Abs–Raspberry-HA (by using a ratio of 3:1 for amphiphile lecithin/anionic carbohydrate). This result may indicate an increase in the solubilization of the distribution system, which translates into an advanced erosion of the outer layer that covers the lipid core. As such, the presence of hyaluronic acid causes an acceleration of the erosion of the NLC coating, with a faster release of the active principle distributed in this external layer. According to a recent study, the co-presence of avobenzone and octocrylene absorbers and herbal extract into lipid nanocarriers assures a decrease of the in vitro release effect of the UV filter. In addition, small amounts of UV absorbers penetrated the collagen membrane [30]. The study demonstrated that the cumulative release of avobenzone and octocrylene from NLC sunscreen after 8 h of analysis was at proportions of 11% and 20%, respectively.

The data showed that NLC-UV Abs-Raspberry with or without carbohydrate surface modifier has a dual capability to protect the included actives from photo-instability phenomena and enable a low penetration efficiency (BMMDBM and EHS will mainly remain located on the surface of the skin). Therefore, these results underline that NLC can drastically reduce the harmful skin permeation of UV absorbers and confirm the high potential of NLC-UV Abs-Raspberry-HA for the new generation of nano-sunscreen formulations.

Table 2 shows the kinetic parameters (rate constant *k*, correlation coefficient *R*^2^, and release coefficient, *n*) obtained by fitting the in vitro release data for EHS and BMDBM with the five kinetic models. For the NLC-UV Abs-Raspberry, the best correlation coefficient *R*^2^ was obtained by the Peppas–Korsmeyer model for both UV absorbers, and the release coefficient *n* < 0.45 indicates the existence of a Fickian diffusion-type mechanism [44]. The best calculated *R*^2^ correlation coefficient for HA–NLC was obtained for the Hixson–Crowell model, indicating that the release is dependent on both diffusion and surface erosion of the hybrid NLC [45]. As previously observed, this behaviour is most likely explained by the presence of the hydrophilic surface modifier that enhances the erosion phenomenon.

### 2.5. In Vitro Determination of Antioxidant Activity

Antioxidant activity (AA) is associated with the prevention of oxidative stress caused by free radicals (reactive oxygen species, ROS) in the human body and characterises the ability of antioxidants to scavenge these free radicals responsible for the oxidation of lipids, proteins, DNA and other molecules.

Antioxidant properties in cosmetic formulation concerns are important due to the safety aspects, such as preventing oxidative stress, inflammation, and damage caused by free radicals and UV radiation. Synthetic UV absorbers are already gaining attraction as skin protection from sunburn, photo-aging, and photo-carcinogenesis [46]. However, the efficacy and safety of most sunscreens’ constituents are hindered by their photostability and damage to marine ecosystems. Herbal plants manifested effective protective mechanisms against the deleterious side effects of oxidative stress and ultraviolet radiation. The production of ROS triggered by UV radiation and intrinsic ageing can subsequently overwhelm the skin’s natural endogenous defences, leading to oxidative damage [47].

**Figure 5:**
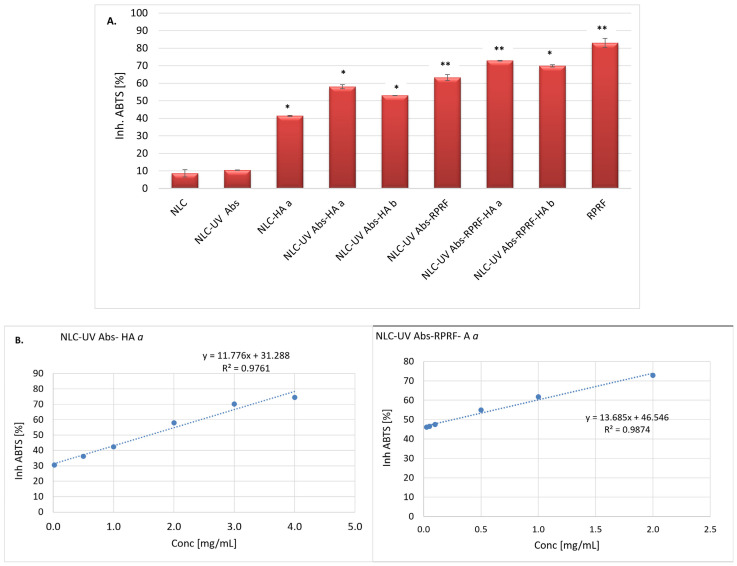
(**A**) Antioxidant activity of conventional NLC and HA-decorated NLC-UV absorbers–raspberry; (**B**) IC_50_ values determined by ABTS assay. All experiments were carried out in triplicate. * *p* < 0.05; ** *p* < 0.005. Data are expressed as mean ± SD, *n* = 3, NLC vs. other groups.

Natural Raspberry polyphenol-rich fraction (RPRF) exerts beneficial skin properties (such as improved skin elasticity, hydration, and skin repairing actions) through their antioxidant activity and the regulation of UV-induced skin inflammation, barrier impairment, and ageing [48]. NLC-UV Abs-Raspberry with and without surface HA modifier showed 72.83% ± 0.17 and 63.2% ± 1.72 free radical scavenging activity as measured by ABTS assay (Figure 5A). There was a significant difference in the percentage of NLC-UV Abs-Raspberry and that of HA decorated NLC, the latter proving a significantly superior antioxidant activity. These findings underscore the impact of hyaluronic acid on the antioxidant ability of entire delivery lipid nanocarriers. The outcomes of Mohammed et al. showed that HA had an effective hydrogen peroxide scavenging activity [41], thus providing a first justification for the improvement of antioxidant activity. Moreover, NLC-HA possessed a moderate antioxidant activity of 41.34% ± 0.29 as compared to NLC without HA (8.62% ± 2.08). This is caused by a confirmed antioxidant potential of HA coupled with the presence of marula oil and coconut butter [49], which can function as secondary antioxidants. The previsible mechanism of these natural lipids is based on reducing the rate of lipid oxidation and free radical scavenger [50].

The improvement of AA% is also due to Raspberry fraction, known as a rich source of polyphenols and anthocyanins. These phytochemicals act as effective antioxidants by their ability to reduce radical species, mainly by donating hydrogen and their stabilization by the π–π electron resonance effect [27]. Similar results were reported by Durgo et al., using ABTS assay in determining polyphenol antioxidant activity [51]. This showed that the presence of phenolics, flavonoids, and proanthocyanidins was highly correlated with free radical scavenging activity, reaching 31.1 ± 0.6%. 

Figure 5B shows the IC_50_ values (the concentration that can inhibit free radicals by 50%) for two representative lipid nanocarriers. The IC_50_ value of NLC-UV Abs-Raspberry-HA, 0.252 mg/mL, reflects greater free radical scavenging ability as compared to that of NLC-UV Abs-HA. This lower IC_50_ shows more potency of Raspberry fraction hyaluronic acids than only HA. The IC_50_ determined for HA-decorated NLC-UV Abs-Raspberry is 6.25-fold lower than NLC-UV Abs-HA (IC_50_ = 1.589 mg/mL), which indicates a more free radical scavenging efficacy. In a related study, Binazir et al. reported SE-loaded NLC with an IC_50_ value of 0.68 mg/mL [52]. In addition, Karimi et al. underlined the advantages of encapsulation influence when comparing the antioxidant activity of turmeric extract loaded NLC with native turmeric extract [43].

The antioxidant efficacy of NLC-UV Abs-Raspberry-HA could be attributed to its phytochemicals (such as phenolic acids, flavonoids, and anthocyanins) and may also be the result of synergistic actions with secondary antioxidants, such as marula oil, coconut butter, and hyaluronic acid. In addition, between the natural and synthetic actives contained in the hybrid HA-decorated NLC (i.e., phytoconstituents in RPRF, lipids in CB, MO and UV absorbers), synergistic and/or additive effects may occur, which manifest themselves by potentiating antioxidant, photoprotective activities, etc. The appearance of additive effects, due to the capacity of each phytoconstituent in RPRF, CB, MO and/or UV absorbers to bind to a single target, respectively, creating a synergistic action (i.e., the actives binding to multiple targets) cannot be excluded. Moreover, the phytoconstituents in the RPRF/CB/MO interact with each other to improve their solubility, and implicitly their bioavailability, which also leads to the occurrence of a synergistic effect.

### 2.6. In Vitro Photoprotective Behaviour and Irradiation Study of Creams Containing Conventional NLC and HA-Decorated NLC-UV Absorbers–Raspberry

**Figure 6:**
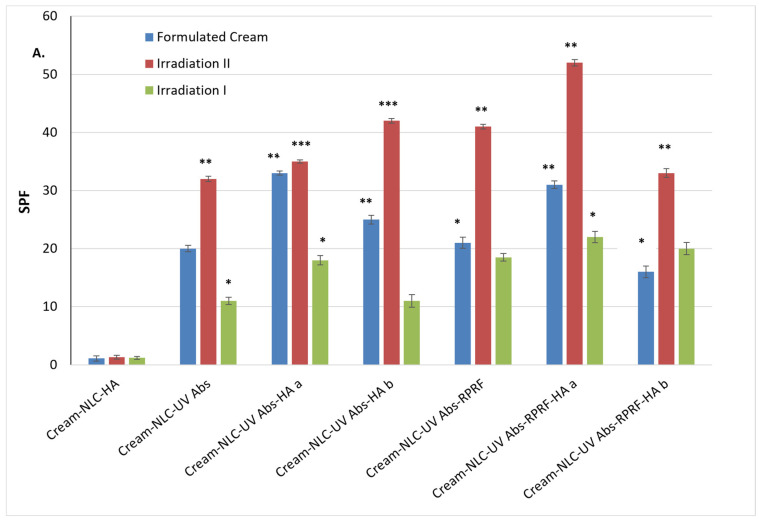

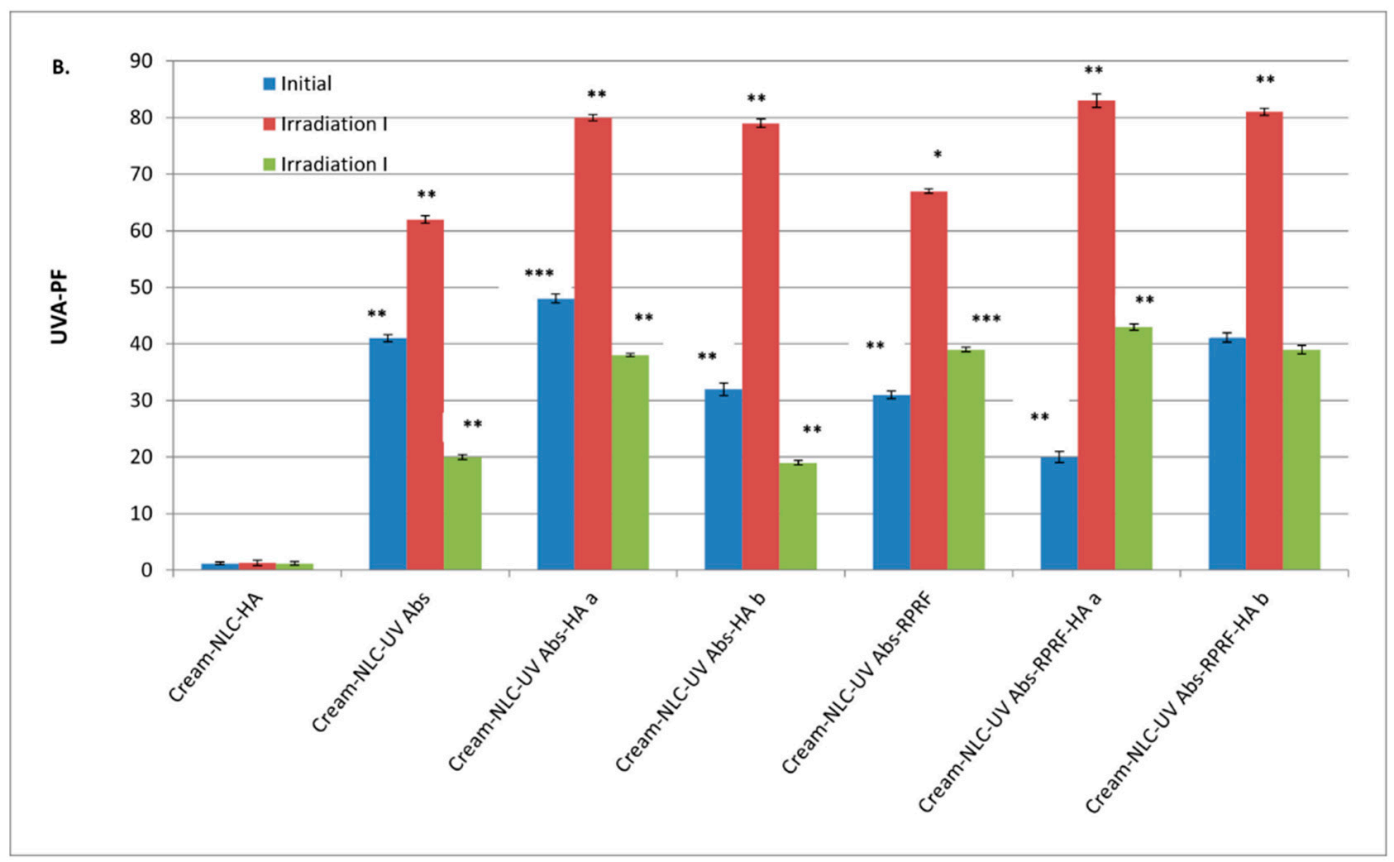
Experimental screening results of in vitro photoprotective potential of various kinds of NLC-based cream, with and without HA: (**A**) SPF values; (**B**) UVA-PF values. All experiments were carried out in triplicate. * *p* < 0.05; ** *p* < 0.005, *** *p* < 0.0005. Data are expressed as mean ± SD, *n* = 3, Cream-NLC-HA vs. other groups.

The findings in topical application of broad-spectrum sunscreens are associated with lowering the risk of premature skin ageing and skin cancer [3]. It is also vital to highlight that. by using the nanoencapsulation of UV absorbers with natural ingredients such as marula oil, coconut wax, and Raspberry polyphenol-rich fractions. it was possible to obtain a photostable sunscreen formulation. From the optimization process, NLC-3 was chosen in the development of topical cream NLC formulations to assess the photoprotection effects of conventional NLC and HA-decorated NLC-UV Absorbers-Raspberry on the skin. All the NLC-cream formulations showed good physical stability, without any coarse particles, and had a homogeneous and consistent texture and appearance (Figure 6), appropriate for topical application.

Photostability impacts sunscreen efficacy, as indicated in the photodegradation of active ingredients in sunscreen formulation, which can be detected by lower SPF values and changes in critical wavelength [3]. In our study, NLC-based cream was used as the sample control to investigate if a synergistic effect could be triggered during the combination of NLC with Raspberry fraction and low concentrations of synthetic UV-Absorber sunscreen formulation.

Sunscreen is defined as broad-spectrum when the critical wavelength at which 90% of the integral spectral absorbance curve between 290 and 400 nm is reached [53]. With a much smaller reduction in the critical wavelength, the developed NLC-based cream presented a broad spectrum of photoprotection with high photostability. The SPF value of NLC-UV Abs-Raspberry-HA a based cream (31 ± 0.66) was significantly higher than that of NLC-UV Abs-Raspberry cream, e.g., 21 ± 0.95 (Figure 6A). A significantly higher SPF value due to the presence of hyaluronic acid suggested a synergistic effect between HA and NLC. The results agreed with the findings of Chu et al. [54] and Niculae et al. [8], which justified the presence of antioxidants to effectively boost the SPF value. In addition, the study of Yap [55] suggested that the formulation with herbal extract provided effective protection against UV-generated ROS and enhanced endogenous antioxidative protection of the skin. Therefore, the application of Raspberry not only enhances the SPF value for the formulation but could also protect the skin against erythema. Plant extracts exhibit a protective ability against UV radiation when used as supplements in sunscreen formulations [14]. In related studies, He et al. observed that polyphenols were excellent ingredients in commercial sunscreen products, possessing protective properties. Velasco et al. developed sunscreen delivery systems for rutin and *plantago lanceolata* extract that exhi-bited high SPF values, i.e., 27.5 ± 2.055 and 28 ± 2.429, when associated with 2% TiO_2_, 2% benzophenone-3, and 7% ethylhexylmethoxy cinnamate [56]. The drastic reduction in the concentration of UV absorbers while maintaining photoprotection greater than 98% represents a major advantage considering the toxicity problems generated by these synthetic UV absorbers. In the same context, solid lipid nanoparticles containing urucum oil and chemical UV filter octyl methoxycinnamate resulted in a steady decline in the concentration of the chemical filters [57]. In our research, the NLC-based cream developed contains only a 5% blend of BMDBM and EHS, 22% functional lipids (marula oil and coconut butter), 0.5% HA, and 3.5% Raspberry polyphenol fraction.

To quantify the photoprotective action of NLC-based creams, they were subjected to a simulated irradiation for 1.5 h at energy 19.5 J/cm^2^, to check the optical stability [58]. The results obtained show that an elementary irradiation process for NLC-BMDBM-EHS-Raspberry with and without HA did not compromise the photoprotective ability of BMDBM and EHS. In the previous study, the photo-instability of BMDBM was reported; BMDBM’s photoprotective properties decreased within 1 h of exposure to sunlight by up to 60% [34]. The explanation is based on the enol tautomer form of BMDBM, which undergoes, upon irradiation, photoisomerization to the diketone tautomer [6]. Thus, UVA efficiency is lost, and the skin can be damaged, i.e., by dermatitis and allergies, due to the dangerous photodegradation products of BMDBM. 

In our study, a significant increase in the photoprotective potential of NLC creams demonstrated high photostability with SPF values higher than 35, e.g., SPF of 32–42 (obtained for NLC-UV Abs with and without HA) and 20–52 (for those obtained by coupling UV-Absorbers with Raspberry fraction). These SPF values led to an impressive amount of absorbed UVB radiation, e.g., they provide >99% protection against UVB. The most effective photoprotection was provided by NLC-based creams with dual content of UV-absorbers and RPRF, the latter showing a 25% increase in SPF value, compared to NLC–UV–Abs with/without HA and without Raspberry fraction content. Other related studies reported an increase of between 5% and 10% in SPF following the combination of DHHB, EHS, and natural hesperidin [35] or naringenin with UVA filter diethyl-amino-hydroxy-benzoyl hexyl-benzoate [59].

The significant enhancement of the photoprotective effect of NLC formulations after short irradiation could be attributed to a combined effect: i. a better interaction of the two BMDBM and EHS absorbers with UV radiation, because of their release from the lipid core matrix upon irradiation; this aspect is translated into improved absorption of UVB and its dissipation in the form of heat. ii. the antioxidant and photoprotective attributes of phytochemicals from RPRF. In addition to the presence of Raspberry in the NLC, which is extremely important for promoting greater stability for BMDBM, cyanidins and delphinidins from raspberries are strongly absorbed in the visible and UV spectrum, with maximum absorbances in the 500–550 and 280–320 nm ranges [60]. Furthermore, the anthocyanins in raspberries, delphinidin-3-glucoside, cyanidin-3-glucoside, petunidin-3-glucoside and malvidin-3-glucoside are known as effective actives for protecting human keratinocytes against damage caused by UV radiation, and can delay skin ageing [61], prevent UVB-mediated oxidative stress [62] and UVA-induced damage to human keratinocytes [63].

Prolonged UV irradiation during a period of 3 h resulted in a decrease by 50% of SPF values, which underlines a drastic photostability decrease, with SPF value lower than 25. The serious impairment of photoprotective attributes is most likely the result of the loss of the absorption capacity of UV-Absorbers, simultaneously to the degradation of polyphenols from RPRF. In a related study, Niculae et al. co-encapsulated the BMDBM filter with a UVB filter, octocrylene, to efficiently photo-stabilize it. When NLC-conditioned cream formulations containing BMDBM (2.5%, *w*/*w*) and OCT (1.0%, *w*/*w*) were subjected to irradiation, photostability was reduced by 32.2% [8].

Regarding UV-A photoprotection (Figure 6B), all the NLC-based creams demonstrated a broad UV-A photoprotective spectrum, with UVA-PF values ranging from 20 to 48. AUVA-PF value higher than 20 assures strong protection against UVA radiation (~83%), while the SPF values lead to an impressive amount of absorbed UVB radiation (99%). After irradiation, photoprotection in the UV-A domain was significantly enhanced, even doubling the UVA-PF factor value (e.g., 81 ± 0.75 in the case of the cream containing NLC-BMDBM-EHS-Raspberry-HA). Regarding the amplification in the UV-A domain, BMDBM is directly responsible, which, through keto-enol tautomerism, guarantees the chemical balance, favouring the enol form, with its ability to establish intramolecular hydrogen bonds [6]. The photoprotective results are remarkable taking into consideration the use of small amounts of synthetic UV filters (2.50 to 2.75% for each solar filter), situated below the limit of European Legislation (maximum 5% of EHS and 10% of BMDBM) [64], the toxicological potential being significantly diminished.

## 3. Materials and Methods

### 3.1. Materials

The surfactants L-α-phosphatidylcholine were purchased from Sigma Aldrich Chemie GmbH (Munich, Germany), while poly-oxy-ethylene-sorbitan monolaurate (Tween 20) was purchased from Merck (Darmstadt, Germany). The lipids, glycerol monostearate (GMS) and coconut butter, were purchased from Cognis GmbH (Monheim am Rhein, Germany) and, respectively, Depal SRL (Bucharest, Romania). The marula oil was purchased from Elemental (Oradea, Romania) with the fatty acid composition 70.0% oleic acid (ω-9), 15.0% palmitic acid, 9.0% stearic acid, and 6.0% linoleic acid (ω-6). The Raspberry extract in powder form was obtained by freeze-drying the fruits for 48 h, at −55 °C. The sunscreen ethyl hexyl salicylate (EHS) was purchased from Aako BV (Leusden Arnhemseweg, The Netherlands) and butyl-methoxy dibenzoyl methane (BMDBM) was purchased from Merck as Eusolex 9020. Gallic acid, Folin-Ciocâlteu reagent, NaCl, anhydrous sodium carbonate, phosphate tampon (PBS), glycerol, triethanolamine (98% purity), and hyaluronic acid sodium salt from Streptococcus equi (MW = 1.5 − 1.8 × 10^6^ Da) and Carbopol 940 (99% purity) were supplied by Sigma Aldrich Chemie GmbH (Munich, Germany).

### 3.2. Methods

#### 3.2.1. Characterization of Raspberry Polyphenol Rich Fraction (RPRF)

The Raspberry Polyphenol Rich Fraction (RPRF) was analysed by high-resolution mass spectrometry using a Fourier Transform–ion cyclotron resonance (FT–ICR) spectrometer, SolariX XR 15T (Bruker Daltonics, Bremen, Germany). The Raspberry fraction sample was introduced by direct infusion and positive ESI ionization, with a sample flow rate of 310 µL/h, a nebulization gas pressure (N_2_) of 1.2 bar at 180 °C, and a flow rate of 4 L/min. The spectra were recorded over a mass range between 46 and 2000 amu at a source voltage. 

#### 3.2.2. Preparation of Sunscreen-Loaded Nanostructured Lipid Carrier

High-pressure homogenization (HPH, 6 homogenization cycles, 420 bar, 3.17 min [65,66], was used to prepare classic NLC and hybrid hyaluronic decorated-NLC co-loaded with UV Absorbers and natural RPRF. Briefly, a lipid pre-emulsion was formed by combining a molten lipid phase (consisting of coconut butter, marula oil, glycerol monostearate, with/without UV absorbers, BMDBM and EHS, respectively) with an aqueous phase containing the surfactant blend (with/without Raspberry polyphenol rich fraction). The concentrations of all ingredients used for the preparation of classic NLC and HA-decorated NLC are included in Table 1. For the preparation of NLC with HA surface mo-difier in 60 mL of NLC aqueous dispersion (40 °C), a volume of 40 mL HA solution (2 mg/mL, for NLC-HA *a*) and 20 mL HA solution (2 mg/mL, for NLC-HA *b*), respectively, was gradually added under continuous stirring (high shear homogenization, 1 min, 10,000 rpm, 40 °C); the NLC systems were kept for 30 min under stirring to complete the decoration of NLC with HA. The aqueous NLC dispersions were lyophilized (0.05 mbar, −55 °C, 54 h), using a Martin Christ Alpha 1–2 LD lyophilization system (Martin Christ, Osterode am Harz, Germany), obtaining different solid formulations of NLC and HA de-corated NLC loaded with UV absorbers and/or RPRF (Table 1).

#### 3.2.3. Particle Size and Zeta Potential Analysis

The evaluation of the mean particle diameter (Zave) and the size distribution (PDI) of lipid nanoparticles was carried out by dynamic light scattering (DLS) using a Zetasizer ZS 90 (Malvern Instruments Inc., Worcestershire, UK), equipped with a solid-state laser (670 nm) at a scattering angle of 90°. Samples were prepared by diluting the lipid nanocarriers with deionized water to obtain an adequate scattering intensity and all the measurements were performed in triplicate at a temperature of 25.0 ± 0.1 °C.

The physical stability of the lipid nanocarriers was quantified by measuring the zeta potential (ξ) using the same tool that used the Helmholtz–Smoluchowski Equation (1):

(1) $$\xi =EM\frac{4\pi \eta }{\varepsilon }$$

where ξ—zeta potential, EM—electrophoretic mobility, η—viscosity of the dispersion medium, and ε—dielectric constant.

NLC dispersions were diluted 1:100 with deionized water and the conductivity was adjusted to 50 µS/cm by adding a solution of 0.9% NaCl.

#### 3.2.4. Spectroscopic Characterization by ATR-FTIR

The IR spectra of solid NLC were recorded using a Perkin–Elmer Spectrum 100 instrument (Perkin-Elmer, Shelton, CT, USA) equipped with a horizontal device for attenuated reflectance and diamond crystal, on a spectral window ranging from 4000 to 650 cm^−1^, and a 4 cm^−1^ resolution.

#### 3.2.5. Entrapment Efficiency and Drug Loading

The entrapment efficiency (EE%) and drug loading (LD%) of the UV filters and Raspberry extract were determined according to the method described by Niculae et al. [8] and were calculated according to the following equations:

(2) $$EE\%=\frac{W_{a}{-W}_{s}}{W_{s}}\times 100$$

(3) $$DL\%=\frac{W_{a}-W_{s}}{W_{a}-W_{s}+W_{L}}\times 100$$

where W_a_ is the weight of the active principle (EHS/BMDBM/Raspberry) added in the nanocarriers, W_s_ is the analysed weight of active in the supernatant, and W_L_ is the weight of lipids added in the nanocarriers.

Each NLC (0.5 g) was dispersed into ethanol (1 mL) and the resulting suspension was centrifuged for 15 min at 13000 rpm, (Sigma 2K15, Osterode am Harz, Germany). The supernatant (containing the un-entrapped OCT and BMDBM) was collected and diluted with ethanol. The obtained solution was evaluated at λ = 305 nm (for EHS) and λ = 356.5 nm (for BMDBM), using a UV-Vis-NIR Spectrophotometer V670 (Jasco, Tokyo, Japan). The calibration curves for EHS are y = 0.0167x + 0.0087, *R*^2^ = 0.9996, with concentrations range 0–50 mg/L and for BMDBM: y = 0.1367x − 0.0021, and *R*^2^ = 0.9998 with concentrations range 0–6 mg/L.

The non-encapsulated Raspberry extract was determined by the quantification of polyphenols extracted in water from a known quantity of lyophilized NLC. The polyphenolic content, expressed as gallic acid equivalents (GAE), was determined by using the Folin-Ciocâlteu method, according to ISO 14502-1:2005 [67]. Therefore, 1 mL of water was added in 0.15 g of NLC or RPRF, gently shaken, followed by the sampling of 0.5 mL of the supernatant. The supernatant was mixed with 2.5 mL Folin-Ciocâlteu reagent 10% (*v*/*v*) and 2 mL solution Na_2_CO_3_ 7.5%. The mixture was allowed to react for 1 h at room temperature in a dark place and was centrifugation at 5000 rpm, for 15 min, using a Sigma 2K15 centrifuge. The absorbance of each sample was recorded at λ = 765 nm in triplicate, using a UV-Vis V670 Jasco spectrophotometer (JASCO, Tokyo, Japan). The number of polyphenols was determined using the gallic acid calibration curve in the concentration range 0–100 mg/L, with the equation 0.0096x + 0.0087, *R*^2^ = 0.9919.

#### 3.2.6. In Vitro Determination of Antioxidant Activity

The antioxidant activity of lipid nanocarriers was evaluated in vitro according to ABTS assay. The cationic radical ABTS^●+^ was generated from the reaction between ABTS solution (7 mM) and K_2_S_2_O_8_ (2.45 mM) after 16 h in dark conditions at 4 °C. The ABTS^●+^ solution was normalized at 734 nm, with an absorbance of 0.700 ± 0.01. The samples were prepared by adding 3 mL of ABTS^●+^ solution to 2 mL NLC solution (5 mg/mL) and the absorbance was measured after 4 min, using ethanol as reference. The blank solution was prepared identically by replacing the volume of the NLC with the ethanol. The scavenging capacity of ABTS^●+^ was calculated as inhibition (%) using the following equation:

(4) $$\%ABTSInh.=\frac{A_{0}-A_{s}}{A_{0}}\cdot 100$$

where A_0_ is the absorbance of the blank and A_s_ is the absorbance of the sample.

#### 3.2.7. Cream Formulations of Representative NLC With and Without HA Surface Modifier

The topical NLC formulations were prepared on a shaker at 500 rpm. The conditioning of NLC in the form of a photoprotective cream prototype was performed according to the method described by Lacatusu et al. [30] using a Carbopol polymer. The optimized NLC-UV Abs-Raspberry with and without HA surface modifier was mixed in Carbopol hydrogel at a ratio of 1:1. The final cosmetic formulations contain 2.5 to 2.75% EHS, 2.5 to 2.75% BMDBM, 22 to 24% functional lipids (marula oil and coconut butter), 0 to 3.5% Raspberry polyphenol rich fraction and 0 to 0.65% hyaluronic acid. For comparative purposes, two sample controls (NLC-based cream with only NLC and HA–NLC, without UV absorbers and Raspberry polyphenol rich fraction) were prepared.

#### 3.2.8. In Vitro Release Study

The UVA and UVB absorber release experiments with the designed NLC, with and without surface modifiers, were carried out using Franz diffusion cells (diffusion area of 0.64 cm^2^, volume of 6 mL, Hanson Research Corporation, Chatsworth, CA, USA). Each topical formulation (250 mg) was applied to the surface of the cellulose membrane (0.1 µm; Whatman, Germany), and the experiments were performed for 24 h. The release medium from the receptor chamber is formed by a blend of ethanol–phosphate-buffered saline pH 5.5, 1:1) and was constantly stirred at 400 rpm and thermostated at 37 °C, in order to simulate the skin temperature. At the set time intervals of the experiment (1, 2, 3, 4, 5, 6, 7, 8, and 24 h), 0.5 mL of each sample was withdrawn from the receptor chamber and was discharged, another 0.5 mL being collected, and diluted with ethanol for quantitative determination. The UV-absorber concentrations were determined by using UV–vis spectrometry at the corresponding wavelengths of the released investigated compounds (see Section 3.2.5).

The release kinetics of UV filters were calculated using the following mathematical model equations: first order: $ln(100-\%R)=k_{1}t$; Higuchi: $\%R=k_{2}\sqrt[{2}]{t}$; Hixson–Crowell: $\sqrt[{3}]{100-\%R}=k_{3}t$, where %R is the percentage of UV filter release at time t; k_1_, k_2,_ and k_3_ are the rate constants for first order, Higuchi and Hixson–Crowell respectively; and n is Please corectthe release exponent. The mathematical models’ equations with the biggest *R*^2^ were selected as the best release kinetic model.

#### 3.2.9. In Vitro SPF and UVA-PF Evaluations—Photostability Studies

The in vitro determination of the main parameters which characterise the anti-UVB and anti-UVA properties, Sun Protection Factor (SPF) and UVA Protection Factor (UVA-PF), were achieved for NLC-based creams (containing from 2 to 2.5% EHS, from 2% to 2.5% BMDBM, from 0.5 to 0.65% HA and from 3 to 3.5% Raspberry rich polyphenol fraction), using a UV–Vis V670 Spectrophotometer Jasco (Jasco, Tokyo, Japan) equipped with integrated sphere and adequate softness (Diffrey and Robson method) [68]. Each NLC-based cream (2 mg/cm^2^) was distributed on an artificial skin membrane (TransporeTM tape), which had been previously placed on a quartz plate, and the UV absorption spectrum was recorded at six different points in the spectral range 290–400 nm; a TransporeTM membrane without sample on the quartz plate was used as a control sample. The SPF and UVA-PF values were calculated using the following equations:

(5) $$\mathrm{SPF}=\frac{\sum_{290}^{400}E_{\lambda }B_{\lambda }}{\sum_{290}^{400}E_{\lambda }B_{\lambda }/MPF_{\lambda }}$$

(6) $$\mathrm{UVA}-\mathrm{PF}=\frac{\sum_{320}^{400}E_{\lambda }B_{\lambda }}{\sum_{320}^{400}E_{\lambda }B_{\lambda }/MPF_{\lambda }}$$

where E_λ_ represents the spectral irradiance of terrestrial sunlight under defined conditions; B_λ_ is the relative erythemal effectiveness; MPF_λ_ is the the monochromatic protection factor for selected wavelength (the difference between the spectrum of measured sample applied on support and support spectrum).

Photostability study. The photostability of NLC-based creams was evaluated by exposing it to UV irradiation using a UV Solar Simulator (BioSun, Vilver Lourmat, France), equipped with a WG320 filter for UVA and UVB spectrum, with an irradiation energy of 19.5 J/cm^2^ for UVA (365 nm) and UVB (312 nm), temperature 31.4 °C. The simulated irradiation was carried out as follows: an initial irradiation (1 h and 30 min on UVA, 365 nm and 2 h and 30 min on UVB, at 312 nm) and a prolonged irradiation (3 h on UVA, 365 nm and 5 h on UVB, at 312 nm).

#### 3.2.10. Statistical Analysis

All measurements were performed in triplicate and expressed as their mean value ± standard deviation (SD). The statistical significance of the experimental data was determined using a one-way analysis of variance (ANOVA) test. Statistical significance was considered at *p* < 0.05.

## 4. Conclusions

An improved version of classic NLC was produced. Natural and functional delivery systems (formulated with marula oil and coconut butter) consisting of carbohydrate-de-corated Nanostructured Lipid Carriers, which co-capture two UV absorbers (BMDBM and EHS) and a Raspberry polyphenol fraction (RPRF), were specifically designed to complement the pharmaco-cosmetic potential, in order to identify a potential synergistic effect. The developed HA-modified NLC had main diameters ranging from 147.3 nm (PDI = 0.157) to 165.6 nm (PDI = 0.173). The HA carbohydrate applied to modify the surface of the lipid nanocarriers maintained electrostatic and steric stability, providing electrokinetic potentials which were more electronegative than −39.5 mV. Quantitative attributes of HA decorated-NLC loaded with the dual UV-Absorbers and RPRF revealed high encapsulation efficiencies, with values between 81% and 92.7% for BMDBM, 80% for EHS, and up to 83% for natural RPRF.

The various creams produced with selected NLC-UV Abs-Raspberry with/without hyaluronic acid indicated that different nanocarriers may prevent the skin damage produced by reactive radical species and enhance the photoprotective potential of synthetic and natural actives. The antioxidant activity of HA modified-NLC, assigned by ABTS assay and IC_50_ values, was superior to conventional NLC. NLC-UV Abs-Raspberry with HA surface modifier inhibits 72.83% ± 0.17 of the harmful cation radicals versus 63.2% ± 1.72 detected for NLC-UV Abs-Raspberry. The IC_50_ value determined for NLC-UV Abs-Raspberry-HA, 0.2523 mg/mL, was 6.25-fold lower than NLC-UV Abs-HA, which reflects the greater free radical scavenging and potency of coupled RPRF-HA compared to NLC-HA.

According to the in vitro release experiments, the covering of NLC with HA accele-rates the release of EHS and almost does not influence the release of BMDBM. In all tested NLCs, the cumulative release of BMDBM remained almost constant, at approximately 2.1%, while the cumulative release of EHS did not exceed 4.6% for NLC-UV Abs-Raspberry-HA, by using a ratio of 3:1 amphiphile lecithin/anionic carbohydrate.

The photoprotection against UV-B radiation, quantified by in vitro determination of SPF, demonstrated an improved protection for developed NLC-based creams, with SPF values ranging from 20 to 52. These SPF values led to an impressive amount of UVB radiation absorbed, thus ensuring more than 99% photoprotection against UVB. The most effective photoprotection was provided by NLC-based creams with dual content of BMDBM, EHS, and Raspberry fraction, the latter showing a 25% increase in SPF value, compared to NLC-UV-Abs with/without HA and Raspberry fraction content. Furthermore, all the NLC-based creams revealed a broad UV-A photoprotective effect, with UVA-PF values varying from 20 to 48. Notable, after a simulated irradiation process, the photoprotection in the UV-A domain was significantly enhanced, a doubling of the UVA-PF factor value, e.g., 81 ± 0.75 being detected for the cream containing NLC-BMDBM-EHS-Raspberry-HA. 

Overall, NLC-UV Abs-Raspberry with hyaluronic surface modifier has multiple capabilities in protecting the included actives from photo-instability phenomena, enabling a low penetration of harmful UV absorber, and providing simultaneously antioxidant and photoprotective efficiency. Moreover, hybrid-NLC can drastically reduce the harmful skin permeation of the synthetic UV-absorbers and confirm the high potential of NLC-UV Abs-Raspberry-HA for the new generation of nano-sunscreen formulations. The HA decorated NLC-conditioned creams might provide a useful platform for developing a sophisticated dermal delivery system for application in topical preparations owing to their favourable moisturizing property, influencing skin permeability, and synergistically imparting antioxidant and photoprotective actions in cosmetic products. 

## Figures and Tables

**Table 1:** Average diameters, polydispersity index, and zeta potential values for hybrid lipid nanocarriers: blank- and loaded-NLC with/without surface HA modifier.

Optimization of NLC Formulations	Composition		Z_ave_ (nm)/PDI /ξ (mV)
	Aqueous Phase	Lipid Phase	
**NLC-1**	Tween 20 (1 g) Lecithin (1 g) Water (88 g)	MSG (3.5 g) Coconut butter (3.5 g) Marula oil (3 g)	Z_ave_ = 184.9 ± 0.55 PDI = 0.219 ± 0.00
			ξ = −55.6 ± 1.25
NLC-1-HA *a*		MSG (3.5 g) Coconut butter (3.5 g) Marula oil (3 g) 2 mg/mL sol. HA (40 mL for ***a*** and 20 mL, for ***b***)	Z_ave_ = 181.8 ± 1.57 PDI = 0.258 ± 0.01
			ξ = −56.6 ± 1.89
NLC-1-HA *b*			Z_ave_ = 188.8 ± 2.00 PDI = 0.252 ± 0.01
			ξ = −50.9 ± 4.97
**NLC-2**	Tween 20 (1.25 g) Lecithin (0.75 g) Water (88 g)	MSG (3.5 g) Coconut butter (3.5 g) Marula oil (3 g)	Z_ave_ = 147.9 ± 2.91 PDI = 0.192 ± 0.01
			ξ = − 48.8 ± 0.60
NLC-2-HA *a*		MSG (3.5 g) Coconut butter (3.5 g) Marula oil (3 g) 2 mg/mL sol. HA (40 mL for ***a*** and 20 mL, for ***b***)	Z_ave_ = 141.7 ± 1.41 PDI = 0.182 ± 0.002
			ξ = −39.7 ± 1.38
NLC-2-HA *b*			Z_ave_ = 149.5 ± 1.89 PDI = 0.192 ± 0.023
			ξ = −40.7 ± 1.05
**NLC-3**	Tween 20 (1.5 g) Lecithin (0.5 g) Water (88 g)	MSG (3.5 g) Coconut butter (3.5 g) Marula Oil (3 g)	Z_ave_ = 139.1 ± 0.95 PDI = 0.164 ± 0.02
			ξ = - 42.7 ± 2.51
NLC-3-HA *a*		MSG (3.5 g) Coconut butter (3.5 g) Marula oil (3 g) 2 mg/mL sol. HA (40 mL for ***a*** and 20 mL, for ***b***)	Z_ave_ = 136.6 ± 1.93 PDI = 0.196 ± 0.005
			ξ= −39.5 ± 0.46
NLC-3-HA *b*			Z_ave_ = 135.1 ± 2.83 PDI = 0.179 ± 0.014
			ξ = −38.7± 2.53
NLC-UV Abs	Tween 20 (1.5 g) Lecithin (0.5 g) Water (88 g)	MSG (3.5 g); Coconut butter (3.5 g); Marula oil (3 g); BMDBM (0.75 g), EHS (0.75 g)	Z_ave_ = 160.0 ± 2.88 PDI = 0.233± 0.007
			ξ = −49.2 ± 1.15
NLC-UV Abs-HA *a*		MSG (3.5 g); Coconut butter (3.5 g); Marula oil (3 g); BMDBM (0.75 g), EHS (0.75 g); 2 mg/mL sol. HA (40 mL for ***a*** and 20 mL, for ***b***)	Z_ave_ = 154.9 ± 1.64 PDI = 0.246 ± 0.005
			ξ = −48.0 ± 1.68
NLC-UV Abs-HA *b*			Z_ave_ = 154.9 ± 2.39 PDI = 0.246 ± 0.004
			ξ = −50.1 ± 1.78
NLC-UV Abs-RPRF	Tween 20 (1.5 g) Lecithin (0.5 g) Water (88 g) RPRF (1 g)	MSG (3.5 g); Coconut butter (3.5 g); Marula oil (3 g); BMDBM (0.75 g), EHS (0.75 g)	Z_ave_ = 166.6 ± 0.93 PDI = 0.187 ± 0.013
			ξ = −43.6 ± 1.11
NLC-UV Abs-RPRF-HA *a*		MSG (3.5 g); Coconut butter (3.5 g); Marula oil (3 g); BMDBM (0.75 g), EHS (0.75 g); 2 mg/mL sol. HA (40 mL for ***a*** and 20 mL, for ***b***)	Z_ave_ = 149.7 ± 3.25 PDI = 0.156 ± 0.001
			ξ = −45.1 ± 1.30
NLC-UV Abs-RPRF-HA *b*			Z_ave_ = 150.3 ± 1.46 PDI = 0.157 ± 0.03
			ξ = −39.2 ± 1.72

**Table 2:** Kinetic data obtained at the controlled release of UV absorbers from NLC.

NLC Formulations	Compounds	Zero Order		First Order		Higuchi		Hixson-Crowell		Peppas–Korsmeyer		
		*R* ^2^	*k*_0_	*R* ^2^	*k*_1_	*R* ^2^	*k*_2_	*R* ^2^	*k*_3_	*R* ^2^	*k*_4_	*n*
NLC-UV Abs-Raspberry	EHS	0.9754	0.4713	0.9751	0.0048	0.9474	1.716	0.9679	0.0005	0.9763	4.1737	0.3443
	BMDBM	0.9426	0.3183	0.9416	0.0032	0.9153	1.285	0.9621	0.0004	0.9864	3.5268	0.3705
NLC-UV Abs-Raspberry HA *a*	EHS	0.9725	0.4848	0.972	0.0049	0.9693	1.951	0.9932	0.0007	0.9641	4.1273	0.3738
	BMDBM	0.9896	0.2573	0.9893	0.0026	0.9766	1.003	0.9984	0.0003	0.9936	2.8583	0.4581
NLC-UV Abs-Raspberry HA *b*	EHS	0.9824	0.4642	0.982	0.0047	0.9778	1.837	0.9950	0.0006	0.9485	3.625	0.4434
	BMDBM	0.9926	0.2596	0.9925	0.0026	0.9842	1.006	0.9992	0.0003	0.9873	2.836	0.4690

## Data Availability

The original contributions presented in the study are included in the article/Supplementary Material, further inquiries can be directed to the corresponding authors.

## Supplementary Materials

The following are available online at https://www.mdpi.com/article/10.3390/ph18010016/s1, Table S1. Calculated mass and measured mass for some compounds from the Raspberry fraction identified by FT-ICR analysis (ESI positive).
