# Supplementary material for: Hybrid Carbohydrate–Lipid Nanocarriers: In Vitro Efficacy Gene-Rated by Association of UV-Absorbers and Raspberry Polyphenols Rich-Fraction
Source: Pharmaceuticals (Basel). 2024 Dec 26;18(1):16. doi: 10.3390/ph18010016 (PMC11769146; doi:10.3390/ph18010016)

Supplementary Materials

Hybrid Carbohydrate-Lipid Nanocarriers: In Vitro Efficacy Gene-Rated by Association of UV-Absorbers and Raspberry Polyphenols Rich-Fraction

Nicoleta Badea[1], Diego Samayoa[1], Alina Moroşan[1], Cristina Ott[1] and Ioana Lacatusu[1,*]

Table S1. Calculated mass and measured mass for some compounds from the raspberry fraction identified by FT-ICR analysis (ESI positive)

**COMPOUND**	**FT-ICR MS (ESI positive)**		
	Molecular formula	Calculated mass (m/z)	Measured mass (m/z)
**Caffeic acid** 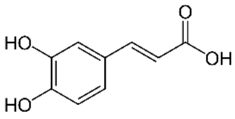	$C_9H_8O_4$	181.0495	181.0497
	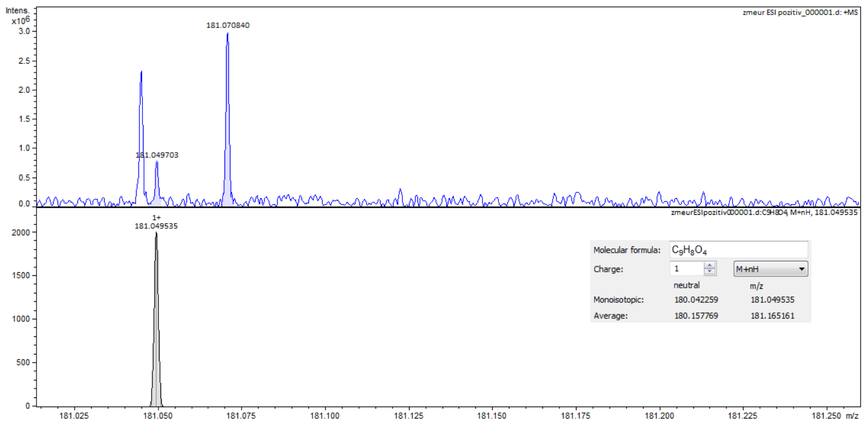		
**Myo-inositol**	Molecular formula	Calculated mass (m/z)	Measured mass (m/z)
	$C_6H_{12}O_6$	181.0706	181.0708
	$C_6H_{12}O_6Na$	203.0526	203.0527

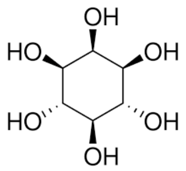

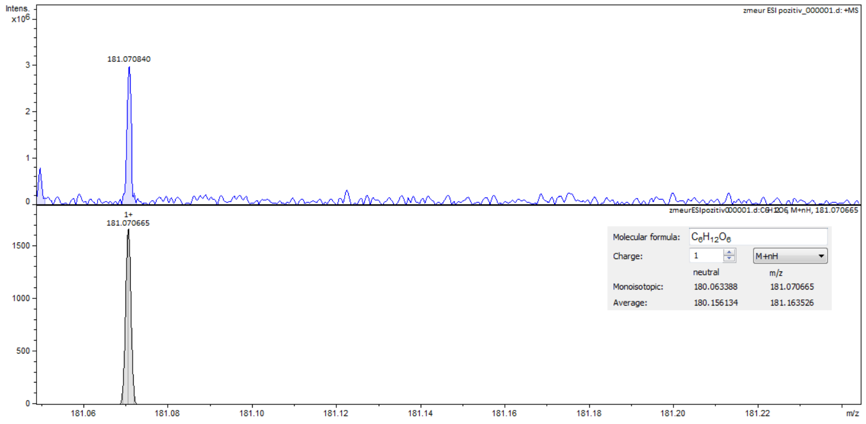

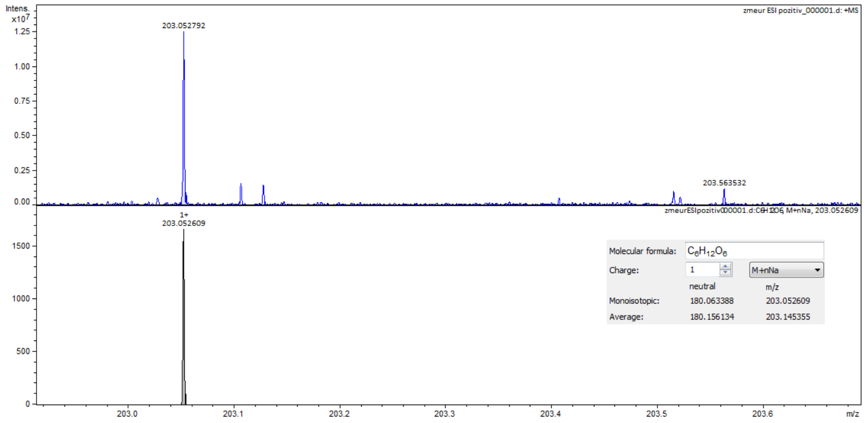

Ferulic acid

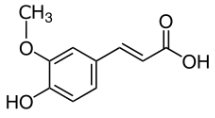

Molecular formula	Calculated mass (m/z)	Measured mass (m/z)
$C_{10}H_{10}O_4$	195.0651	195.0652
$C_{10}H_{10}O_4Na$	217.0471	217.0481

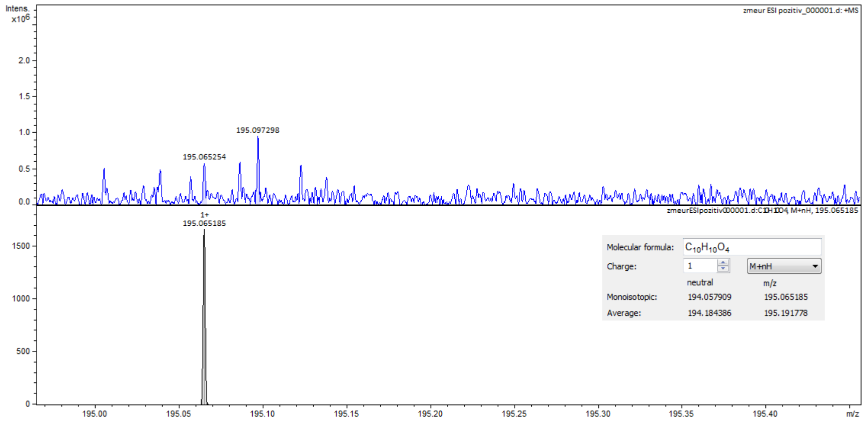

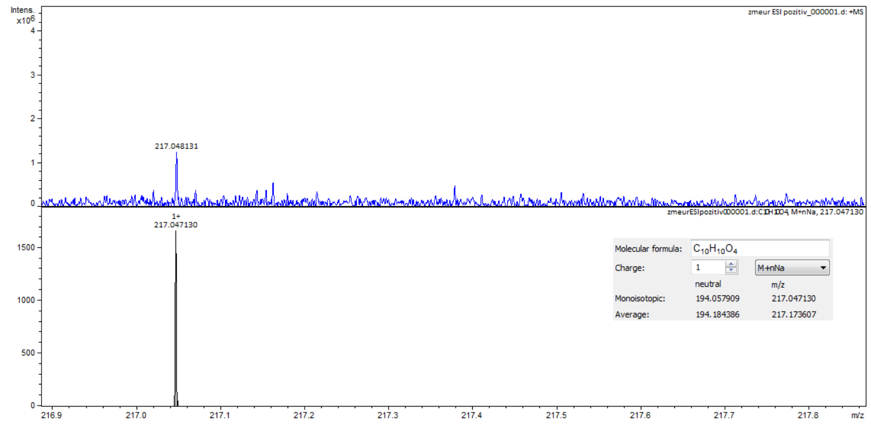

Chlorogenic acid

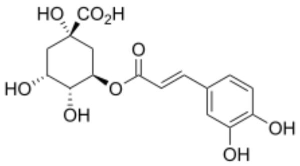

Molecular formula	Calculated mass (m/z)	Measured mass (m/z)
$C_{16}H_{18}O_9Na$	377.0843	377.0844

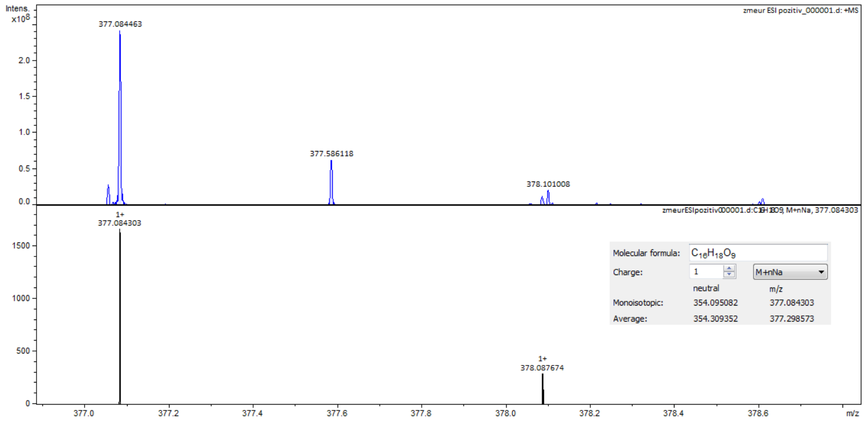

Sinapic acid

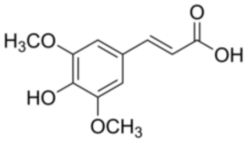

Molecular formula	Calculated mass (m/z)	Measured mass (m/z)
$C_{11}H_{12}O_5$	225.0757	225.0759
$C_{11}H_{12}O_5Na$	247.0576	247.0578

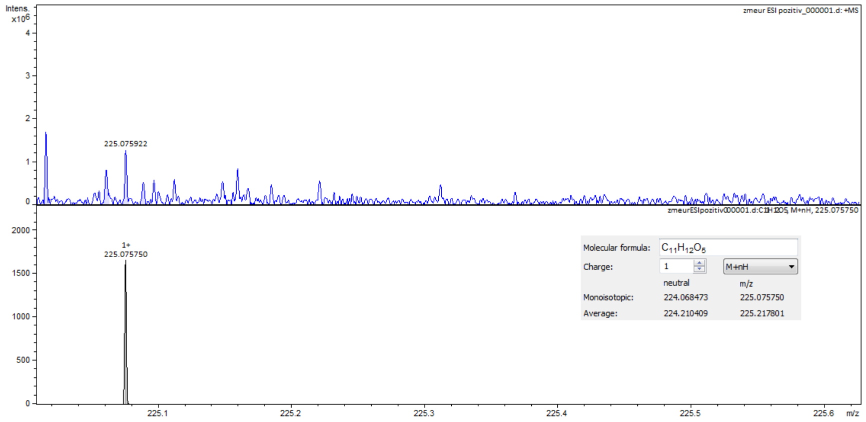

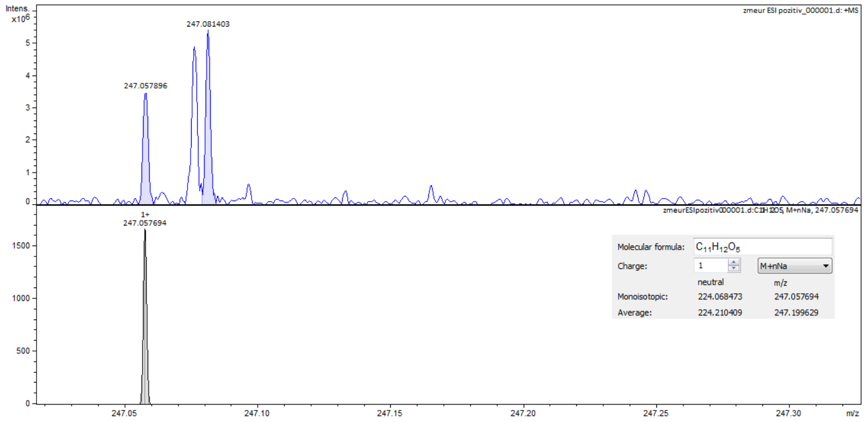

Catechin

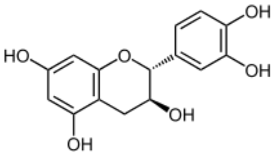

Molecular formula	Calculated mass (m/z)	Measured mass (m/z)
$C_{15}H_{14}O_6$	291.0863	291.0863

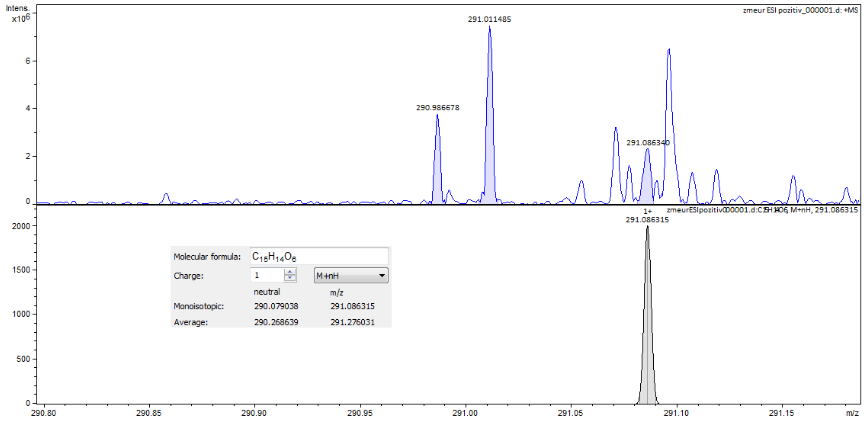

Cyanidin-3-glucoside (Chrysanthemin)

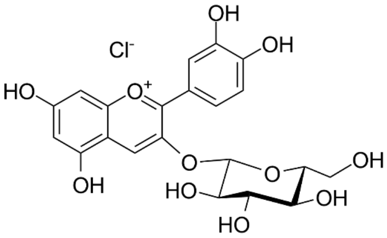

Molecular formula	Calculated mass (m/z)	Measured mass (m/z)
$C_{21}H_{21}O_{11}^{+}$	449.1078	449.1080

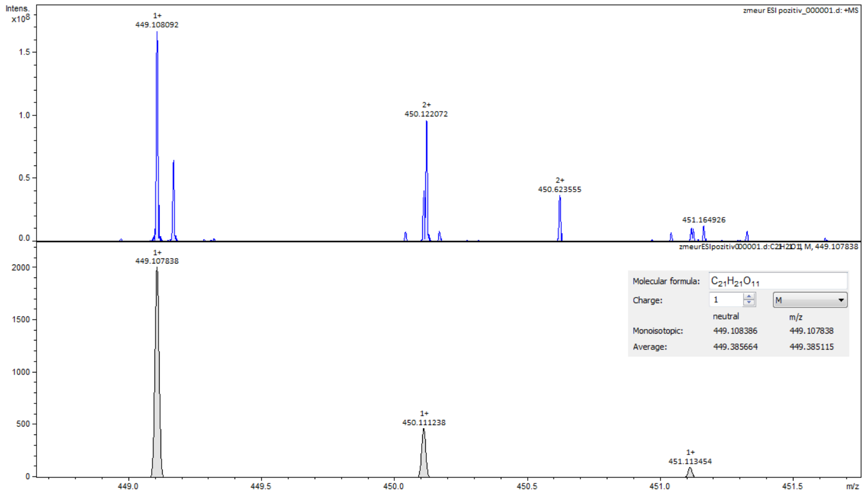

Cyanidin 3-rutinoside (Antirrhinin):

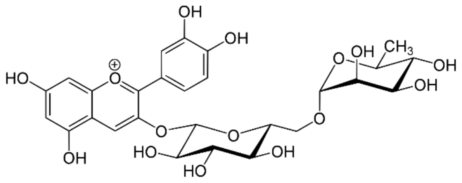

Molecular formula	Calculated mass (m/z)	Measured mass (m/z)
$C_{27}H_{31}O_{15}^{+}$	595.1657	595.1661

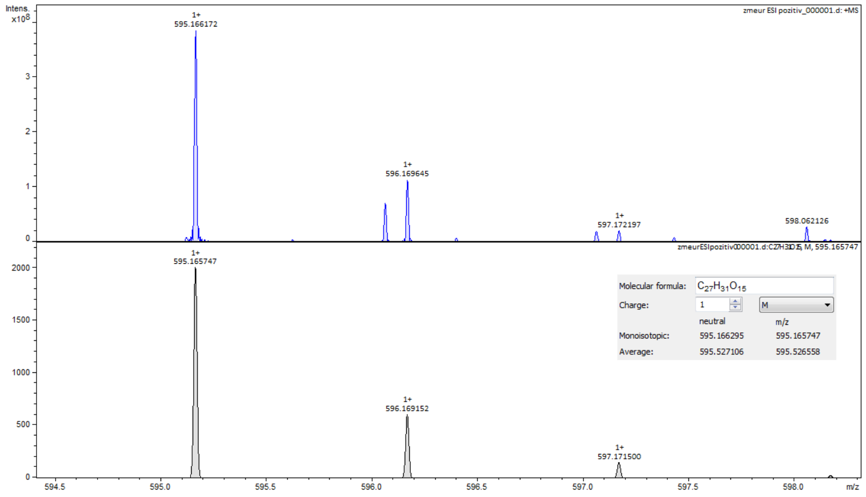

Delphinidin 3-glucoside (Myrtillin):

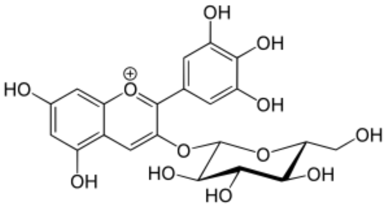

Molecular formula	Calculated mass (m/z)	Measured mass (m/z)
$C_{21}H_{21}O_{12}^{+}$	465.1027	465.1091

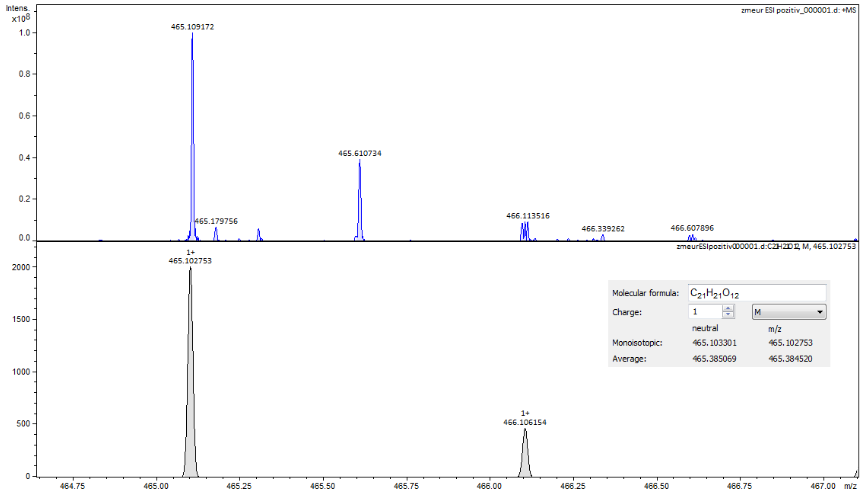

Delphinidin 3-O-rutinoside (Tulipanin)

Molecular formula	Calculated mass (m/z)	Measured mass (m/z)
$C_{27}H_{31}O_{16}^{+}$	611.1606	611.1609

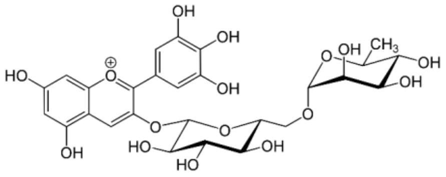

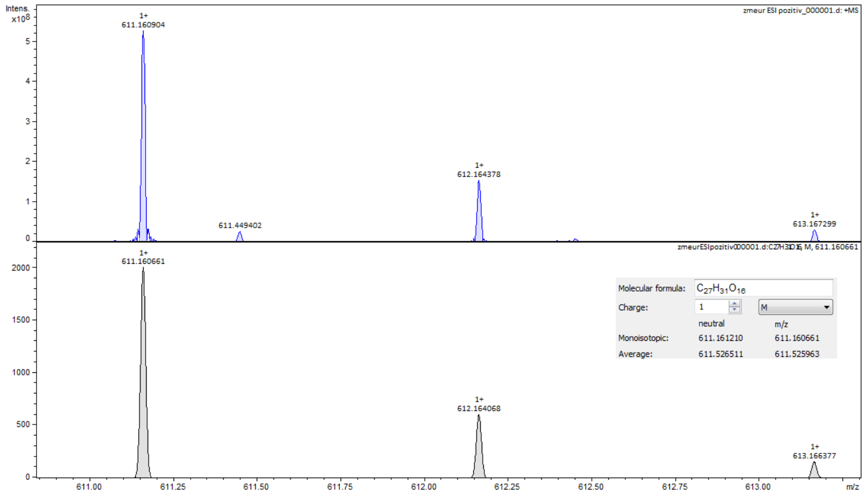

Quercetin 3-O-glucuronide (Miquelianin)

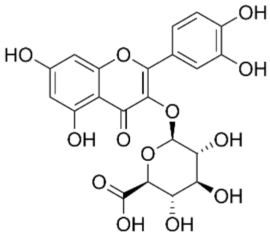

Molecular formula	Calculated mass (m/z)	Measured mass (m/z)
$C_{21}H_{18}O_{13}$	479.0820	479.0795

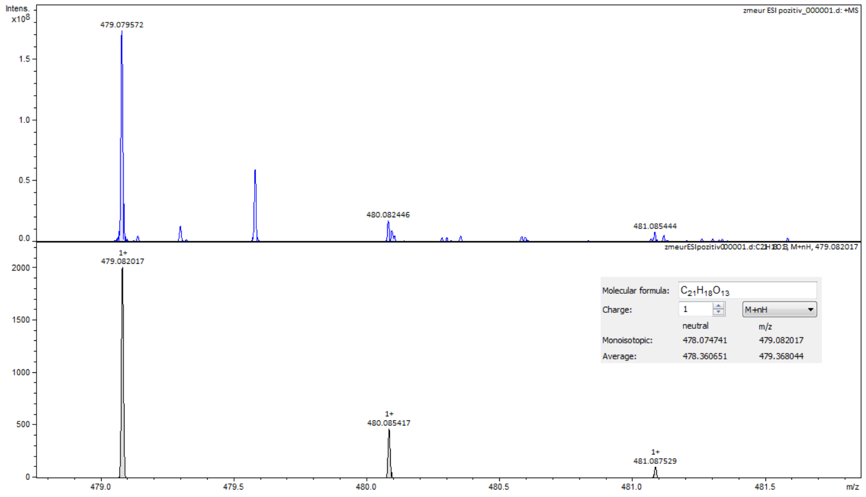

Supplement: Supplementary file 1 [file pharmaceuticals-18-00016-s001.zip › pharmaceuticals-3379845-supplementary.pdf]
